# USP2-45 Is a Circadian Clock Output Effector Regulating Calcium Absorption at the Post-Translational Level

**DOI:** 10.1371/journal.pone.0145155

**Published:** 2016-01-12

**Authors:** Daniel Pouly, Sébastien Chenaux, Virginie Martin, Maja Babis, Rafael Koch, Emi Nagoshi, Vladimir L. Katanaev, Frédéric Gachon, Olivier Staub

**Affiliations:** 1 University of Lausanne, Department of Pharmacology and Toxicology, Rue du Bugnon 27, CH-1005 Lausanne, Switzerland; 2 University of Geneva, Department of Genetics & Evolution, 30, Quai Ernest Ansermet, CH-1211 Geneva, Switzerland; 3 Department of Diabetes and Circadian Rhythms, Nestlé Institute of Health Sciences, CH-1015 Lausanne, Switzerland; 4 National Centre of Competence in Research “Kidney.ch”, Zurich, Switzerland; University of Lübeck, GERMANY

## Abstract

The mammalian circadian clock influences most aspects of physiology and behavior through the transcriptional control of a wide variety of genes, mostly in a tissue-specific manner. About 20 clock-controlled genes (CCGs) oscillate in virtually all mammalian tissues and are generally considered as core clock components. One of them is Ubiquitin-Specific Protease 2 (*Usp2*), whose status remains controversial, as it may be a cogwheel regulating the stability or activity of core cogwheels or an output effector. We report here that *Usp2* is a clock output effector related to bodily Ca^2+^ homeostasis, a feature that is conserved across evolution. *Drosophila* with a whole-body knockdown of the orthologue of *Usp2*, CG14619 (d*Usp2*-kd), predominantly die during pupation but are rescued by dietary Ca^2+^ supplementation. *Usp2*-KO mice show hyperabsorption of dietary Ca^2+^ in small intestine, likely due to strong overexpression of the membrane scaffold protein NHERF4, a regulator of the Ca^2+^ channel TRPV6 mediating dietary Ca^2+^ uptake. In this tissue, USP2-45 is found in membrane fractions and negatively regulates NHERF4 protein abundance in a rhythmic manner at the protein level. In clock mutant animals (*Cry1/Cry2*-dKO), rhythmic USP2-45 expression is lost, as well as the one of NHERF4, confirming the inverse relationship between USP2-45 and NHERF4 protein levels. Finally, USP2-45 interacts *in vitro* with NHERF4 and endogenous Clathrin Heavy Chain. Taken together these data prompt us to define USP2-45 as the first clock output effector acting at the post-translational level at cell membranes and possibly regulating membrane permeability of Ca^2+^.

## Introduction

All organisms that undergo day/night cycles imposed by the rotation of earth have to anticipate these changes to consequently adapt their physiology and behavior. Throughout evolution, they have thus acquired a circadian clock to generate biological rhythms with a period of approximately 24 hours. In mammals, a central clock located in the suprachiasmatic nucleus (SCN) of the hypothalamus is reset daily by sunlight and coordinates slave peripheral clocks in virtually all cells (reviewed in [1]). At the molecular level, the circadian clock relies on interconnected transcriptional and translational feedback loops. Briefly, BMAL1 heterodimerizes with CLOCK or NPAS2 and drives the expression of target genes including the repressors *Per1/2* and *Cry1/2* [2–4]. PER1/2 and CRY1/2 accumulate, repress gene expression including their own [5, 6] and are finally degraded by the proteasome, allowing a new cycle to start. Gene expression profilings performed in the SCN and various peripheral tissues revealed that around 10 to 20% of the known genes have a rhythmic expression. However, clock-controlled genes (CCGs) are essentially tissue-specific [7–9], but less than 20 genes including *Usp2* meet the criteria of rhythmic expression in all studied tissues [9, 10]. They can be separated into 2 categories: the core clock cogwheels and the output effectors. Namely, core clock cogwheels were considered so since their mutation or inactivation leads to obvious defects in circadian behavior (reviewed in [11]). Oppositely, the output effectors do not play a role in the ticking of the molecular oscillator but participate in regulation of gene expression in various physiological processes, as exemplified by the transcription factors of the PARbZip family [12, 13] or KLF15 [14, 15]. In contrast to these transcriptional regulators, *Usp2* encodes the well conserved deubiquitylating enzyme (DUB) USP2 that is involved in post-translational regulation of protein function and stability by Ubiquitin (Ub) and its relatives the Ubiquitin-like (Ub-like) proteins SUMO, NEDD8 and ISG15 [16–19].

The murine *Usp2* gene encodes two protein isoforms: USP2-45, which is clock-controlled and widely if not ubiquitously expressed among mammalian tissues and USP2-69, which is clock-independent and mainly found in heart, testis and skeletal muscle [4, 16, 20–25]. Up to date, USP2-45 is the only known body-wide, clock-controlled and rhythmic DUB [7–9] and was suspected to regulate the stability of core clock cogwheels (reviewed in [26]). In addition, the inactivation of *Usp2* in two independent total knockout mouse models revealed alteration of circadian functions in terms of light-induced phase resetting and controversial data on increased free-running period [24, 27–29]. Besides this disappointing situation in the SCN, USP2 seems to play roles in several peripheral organs. Indeed, the independent characterization of 3 different *Usp2*-KO mouse models pointed out the implication of *Usp2* in male fertility, hepatic gluconeogenesis and possibly peroxisome function [22, 23, 30].

Bodily Ca^2+^ homeostasis in mammals is a tightly regulated process maintaining circulating Ca^2+^ within its physiological range and disruption of this equilibrium can lead to several pathological conditions such as cardiac failure nervous system dysfunction or osteoporosis. The balance is ensured by the interplay between dietary absorption in the small intestine, bone formation and resorption and renal reabsorption and the relative transport activities of these three tissues are regulated by the endocrine system (for a review, see [31]).

In the present study, we aimed to disambiguate the circadian role of mUSP2-45 by taking advantage of gene orthology. We first addressed the question whether the controversial circadian status of *Usp2* may be deciphered thanks to functional conservation across evolution from *Drosophila* to mouse. In summary, we found that the inactivation of *Usp2* in mouse and of its orthologue CG14619 in *Drosophila* does not affect the circadian free-running period, but impairs bodily Ca^2+^ homeostasis in both species, especially in dietary Ca^2+^ absorption in mouse small intestine. We subsequently identified the PDZ-domain containing scaffolding protein NHERF4, a known regulator of the intestinal Ca^2+^ channel TRPV6 [32] as a molecular target of USP2-45 in this tissue. *In vitro*, USP2-45 interacts with and likely forms a complex with NHERF4 and Clathrin Heavy Chain (CLH), indicating a function at the cell membrane. *In vivo*, biochemical studies in WT, *Usp2*-KO and clock defective *Cry1/Cry2*-dKO mouse small intestine indicate that USP2-45 negatively regulates the abundance of NHERF4 in membrane fractions at the protein level and in a rhythmic manner. We therefore propose a new model of the mammalian clock in which USP2-45 is a non-transcriptional effector that rhythmically regulates plasma membrane protein dynamics and potentially permeability of Ca^2+^.

## Results

### *Usp2* and its fly orthologue both essentially act as clock output effectors

**Fig 1:**
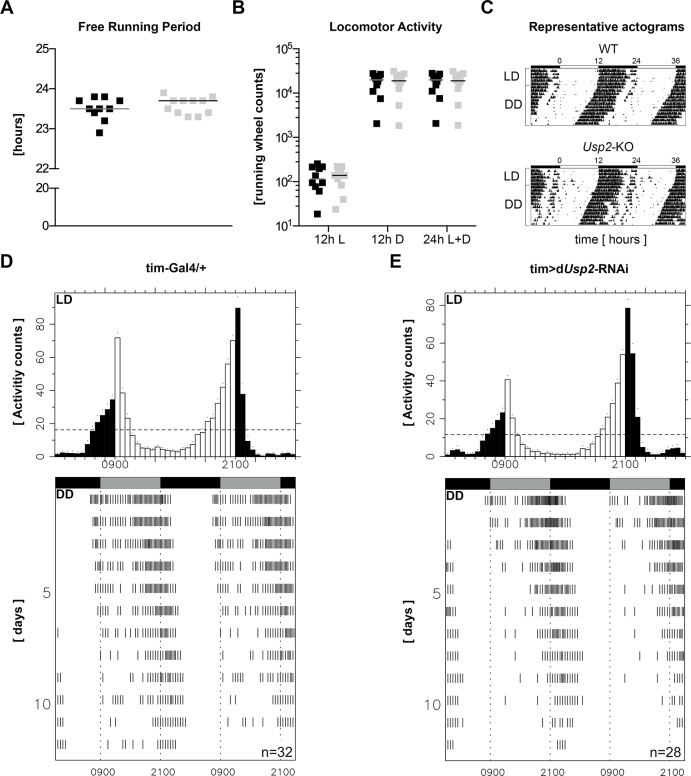
Inactivation of m*Usp2* does not affect the circadian endogenous free-running period. **A:** Mice were housed in individual cages equipped with a running-wheel and entrained for 2 weeks in 12h Light:12h Dark cycles (LD) before being released in constant darkness (DD) for 3 weeks. Endogenous free-running period was calculated by Fourier’s analysis. Data are presented as individual values. Black dots: WT; Gray dots: *Usp2*-KO, bar = median, n = 10–11 animals. **B:** Locomotor activity in light phase (12h L), dark phase (12h D) and over 24 hours (24h L+D) measured by running-wheel counts was plotted as individual values. Black dots: WT; Gray dots: *Usp2*-KO, bar = median, n = 10–11 animals. **C:** 5 days of LD and 12 days of DD are shown on representative actograms of WT and *Usp2*-KO mice. **D**, **E:** The rhythmic activity patterns of control (**D**) and clock neurons-specific d*Usp2*-knockdown (**E**) were studied for 3 days in LD and 12 days in DD. Activity counts were measured during 3 consecutive days in LD. Data are plotted as mean ± standard deviation (SD) of each 30 minutes time interval. Light phase: white bars, dark phase: black bars, dot = SD. Fly activity during 12 days of DD is presented as a representative actogram of each genotype. Subjective day and night periods are indicated above the actogram in grey and black, respectively.

**Fig 2:**
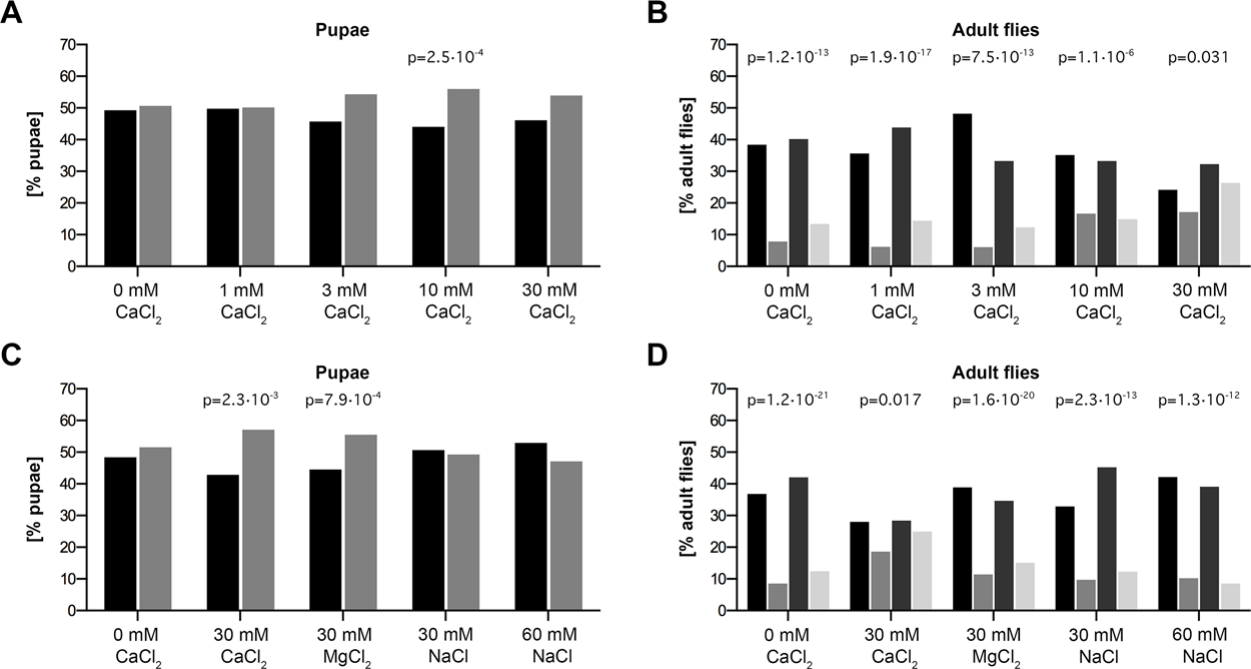
*Usp2* clock output effector status and role in bodily Ca^2+^ homeostasis are conserved across evolution. d*Usp2*-kd and control siblings were bred and raised on the indicated dietary conditions. Pupal cases and adult flies were counted and Mendelian proportions were calculated on the following numbers of pupal cases / adult flies, respectively: 0 mM CaCl_2_: 611 / 266, 30 mM CaCl_2_: 468 / 236, 30 mM MgCl_2_: 924 / 378, 30 mM NaCl: 306 / 155, 60 mM NaCl: 255 / 128 (**A, B**), 0 mM CaCl_2_ (H_2_O): 649 / 164, 1 mM CaCl_2_: 492 / 194, 3 mM CaCl_2_: 473 / 114, 10 mM CaCl_2_: 919 / 174, 30 mM CaCl_2_: 497 / 99 (**C, D**). Pupae: black: control, gray: d*Usp2*-kd. Adult flies: black: control males, gray: d*Usp2*-kd males, dark gray: control females, light gray: d*Usp2*-kd females. Data were tested for equiproportionality and Chi-square test associated p-value is reported when the difference is statistically significant.

We first identified CG14619 (d*Usp2*) as the fly orthologue of mUSP*2* by pBLAST, in accordance with data on human USP2 [33]. Primary analysis identified 4 mammalian homologues for CG14619, namely USP2, USP8, USP21, and USP50. We then compared in more details the three hallmarks and conserved domains of mammalian USPs, specifically the Cys Box, the QDE Box and the His Box [34, 35]. As shown in S1 Fig, USP2 is the closest relative to CG14619 in all three cases. Additionally, USP21, a paralogue of USP2 in vertebrates [36], comes in the second position. We studied the free-running period in mouse and *Drosophila* models lacking *Usp2*. In our data, *Usp2*-KO mice [25] do not display alteration of circadian free-running period (Fig 1A) and have unaltered overall locomotor activity (Fig 1B and 1C). This is contradictory with the data of the Cermakian and Besharse labs, who reported slight alterations on free-running period and locomotor activity, respectively [24, 27]. Similarly to m*Usp2*, CG14619 is a clock-controlled gene displaying rhythmic expression and binding of dCLK, the orthologue of mCLOCK in its promoter region [37, 38] and likely assumes important functions in various tissues. Indeed, whole body knockdown of all CG14619 isoforms (tub>UAS d*Usp2*^KK108078^ RNAi) leads to shortened lifespan and slow locomotor activity (d*Usp2*-kd are actually dying off in the course of experiment, see the progressive reduction of locomotion on individual actograms provided in S1 and S2 Files [33]). We therefore characterized the circadian phenotype of flies lacking CG14619 specifically in clock neurons (tim>UAS d*Usp2*^KK108078^ RNAi) and found no alteration of circadian free-running period (Table 1, Fig 2A and 2B). Together, these data suggest that CG14619 essentially acts as an output effector of the fly clock.

**Table 1:** Inactivation of CG14619 in *Drosophila* does not affect circadian free-running period. Adult flies lacking CG14619 in the whole body (tub>UAS CG14619^KK108078^ RNAi) or only in clock neurons (tim>UAS CG14619^KK108078^ RNAi) and their respective controls were entrained for 3 days in LD at 25°C before being released in constant darkness (DD) for 10 days for calculation of their free-running period. Data are presented as mean ± SD of the indicated number of individuals.

	Genotype	Total flies [n]	Rhythmic flies [%]	FRP [hours]
WT control	w1118	32	90.6	23.9 ± 0.34
tim-Gal4	tim-Gal4/+	32	81.3	24.4 ± 0.28
tub-Gal4	tub-Gal4/+	31	71.0	23.5 ± 0.33
UAS-d*Usp2*^KK108078^ RNAi	UAS-d*Usp2*^KK108078^ RNAi/+	32	84.4	23.6 ± 0.40
tim>UAS-d*Usp2*^KK108078^ RNAi	tim-Gal4/UAS- d*Usp2*^KK108078^ RNAi	27	88.9	24.5 ± 0.36
tub>UAS-d*Usp2*^KK108078^ RNAi	UAS- d*Usp2*^KK108078^ RNAi/+; tub-Gal4/+	7	0	n.d

### *Usp2* participates in bodily Ca^2+^ homeostasis in both mouse and *Drosophila*

**Fig 3:**
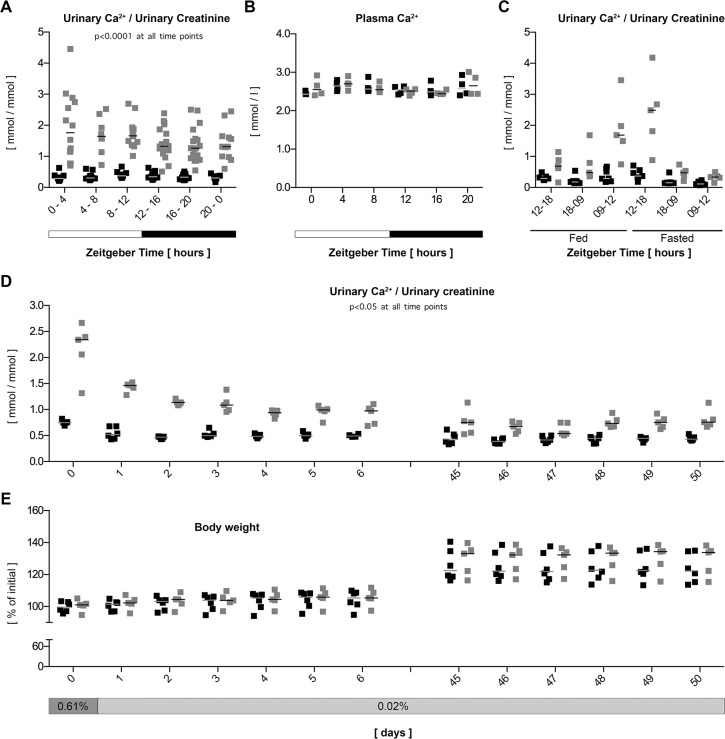
*Usp2*-KO mice have hyperabsorptive hypercalciuria. **A:** Mice were housed in individual metabolic cages and urine was collected every four hours around the clock. Ca^2+^ excretion was calculated as a ratio to creatinine. **B:** Plasma was collected every four hours around the clock and total Ca^2+^ was measured. **C:** Mice were housed in individual metabolic cages with ad libitum feeding or fasted. Urine was collected at the end of the indicated time periods and Ca^2+^ excretion was calculated as a ratio to creatinine. **D, E**: Mice housed in individual metabolic cages were fed a 0.61% Ca^2+^ control diet and were then switched to a 0.02% Ca^2+^ diet for 6 days. The same animals were then tested again after 45 days of 0.02% Ca^2+^ diet for 6 more days. Metabolic parameters were measured every 24 hours and Ca^2+^ to creatinine ratio and body weight were plotted. Data are presented as individual values. Black dots: WT, gray dots: *Usp2*-KO, bar = median, n = 12–18. (**A**), n = 4 (**B**), n = 5–7 (**C, D, E**).

In urine samples obtained from our previous study on *Usp2*-KO mice [25], we serendipitously observed a strong increase in Ca^2+^ excretion in all 4-hour periods studied around the clock (Fig 3A) without any change in plasma Ca^2+^ concentration (Fig 3B). The amplitude of the phenotype is strongly reduced by fasting and dietary Ca^2+^ restriction (Fig 3C–3E), which is characteristic of absorptive hypercalciuria [39]. Furthermore, *Usp2*-KO mice handle long-term reduction of dietary Ca^2+^ intake without loss of body weight (Fig 3E), lowering of plasma Ca^2+^ (Figures A and B in S2 Fig) or compensatory osteoporosis (S1 and S2 Tables). This deregulation is likely restricted to Ca^2+^ transport since Mg^2+^ and PO_4_^3-^, which share transepithelial paracellular transport and hormonal regulation pathways with Ca^2+^, respectively are not affected (Figures C and D in S2 Fig). Additionally, circulating levels of the calciotropic hormones parathyroid hormone (PTH) and Vitamin D are not affected in both standard and Ca^2+^-deprived conditions (Figures A to F in S3 Fig). Finally, expression levels of their target genes [40–42] are not affected in *Usp2*-KO mice as evidenced by our renal transcriptome analysis (NCBI-GEO accession: GSE43517, [25]) and qPCR data on both kidney and small intestine (Figures G and H in S3 Fig). These data demonstrate that the hypercalciuria observed in *Usp2*-KO mice is mainly a consequence of intestinal hyperabsorption, taking place independently of dysfunctions in calciotropic hormones levels and/or signaling cascades. USP2-45 is the main if not the only USP2 isoform expressed in the small intestine since USP2-69 was not detectable in this tissue, but was in heart and skeletal muscle, where its mRNA is present (S4 Fig, [16, 43]). In *Drosophila*, d*Usp2*-kd predominantly die during pupation (Fig 3), but can be rescued by addition of CaCl_2_ to their culture medium in a dose-dependent manner (Fig 3B). The same amount of other cation-chloride salts has no rescuing effect, indicating that Ca^2+^ is indeed critical and that neither Cl^-^ nor osmolality are involved (Fig 3D). Together, these data suggest a conserved function of *Usp2* in bodily Ca^2+^ homeostasis.

### NHERF4 is strongly up-regulated in the small intestine of *Usp2*-KO mice

We next investigated the role of USP2-45 in mouse small intestine to better characterize its action as a clock output effector. To identify its targets in this tissue, we carried out a quantitative proteomic analysis, which revealed a strong up-regulation of the membrane scaffold protein NHERF4 (encoded by the *Pdzd3* gene), being the only major alteration of the detectable proteome (S5 Fig). Interestingly, NHERF4 is a known regulator of the Ca^2+^ channel TRPV6 [32] that mediates the entry of dietary Ca^2+^ from the intestinal lumen into the enterocyte in the course of transepithelial transcellular Ca^2+^ absorption (reviewed in [44]), which is nicely in line with the absorptive hypercalciuria we report.

### USP2-45 interacts with NHERF4 and Clathrin Heavy Chain (CLH)

**Fig 4:**
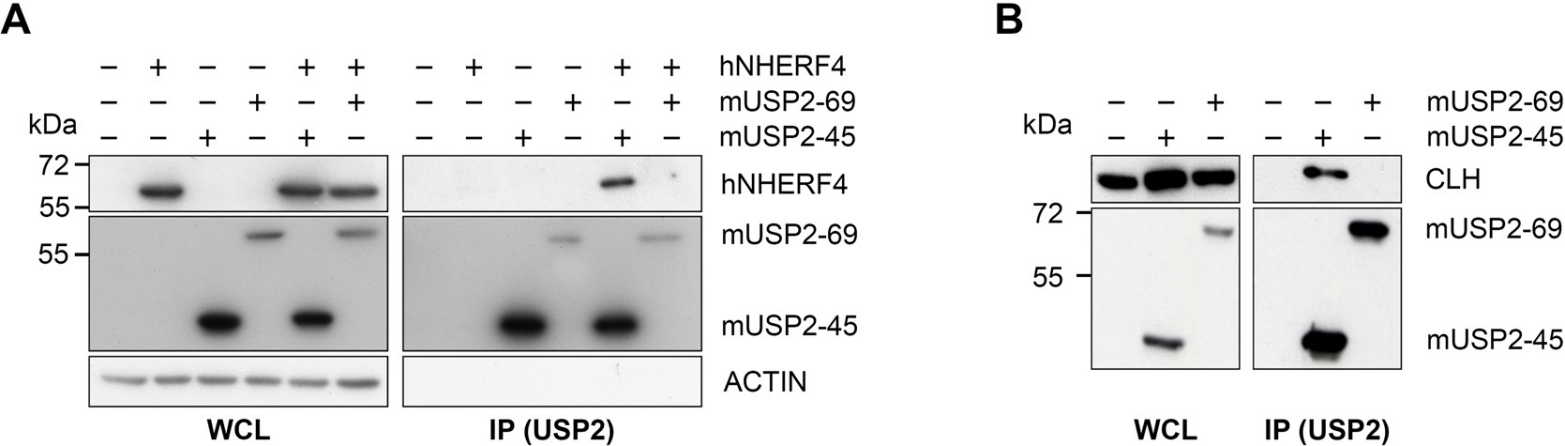
USP2-45 interacts with NHERF4 and endogenous Clathrin Heavy Chain (CLH) in HEK293 cells. HEK293 cells were transfected as indicated and S-tagged USP2-45 or USP2-69 were precipitated with biotinylated S-Protein and Streptavidin-Sepharose beads. Whole cell lysate (left panel, WCL) and precipitation fraction (right panel, IP S-Protein (USP2)) were analysed by SDS-PAGE and western blotting with S-protein HRP (USP2), anti-NHERF4 (**A**) and anti-CLH (**B**) antibodies. Data are western blots from one representative experiment out of three.

To further validate our proteomic result, we co-expressed mUSP2-45, mUSP2-69 and hNHERF4 in HEK293 cells. We observed that USP2-45 interacts with NHERF4 (Fig 4A) and endogenous Clathrin Heavy Chain (CLH) (Fig 4B), whereas the non-circadian isoform USP2-69 does not. These data indicate that USP2-45 is possibly part of a membrane complex regulating surface proteins dynamics.

### NHERF4 is rhythmically and negatively regulated by USP2-45

**Fig 5:**
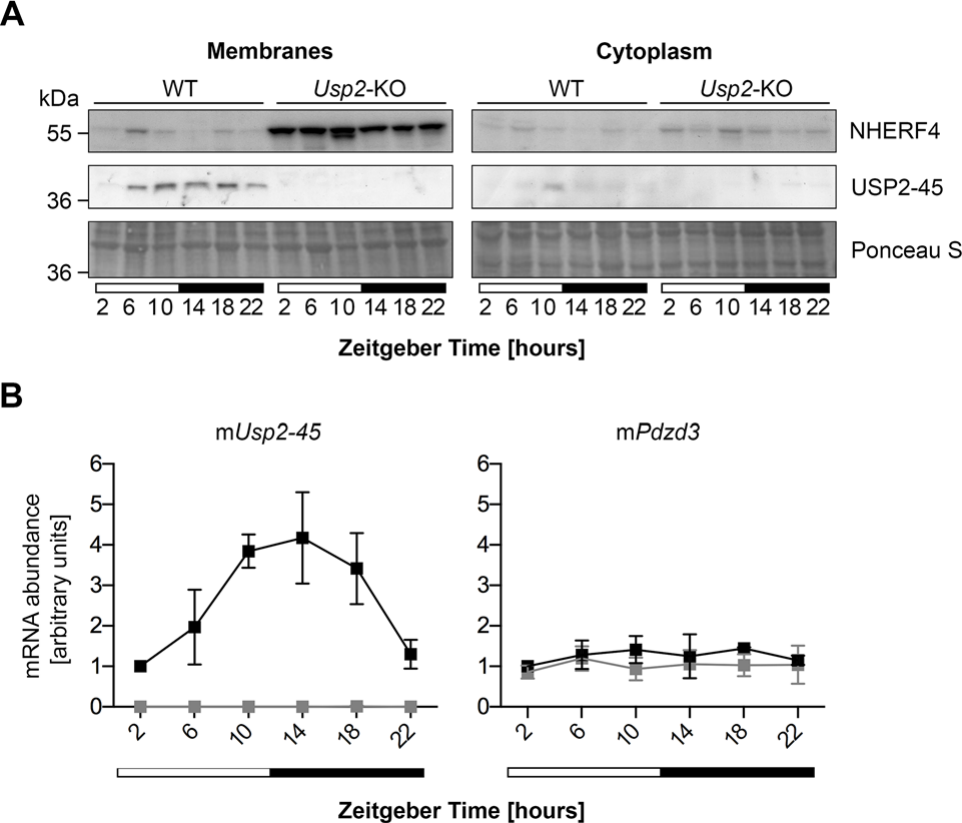
The lack of USP2-45 leads to a loss of NHERF4 rhythm at the protein level in the membrane compartments of small intestine mucosa. **A**: Mucosa of the whole small intestine was dissected at the indicated time points around the clock and membrane and cytoplasmic protein extracts were prepared from the remaining tissue. Protein extracts were analysed by immunoblotting with anti-USP2-45 N-term and anti-NHERF4 antibodies. Ponceau-Red S staining of the membrane was used as loading control. Data are western blots from one representative series of mice out of three. **B**: RNA was extracted from mid-jejunum and the mRNA levels of the corresponding proteins were analysed by semi-quantitative real-time PCR (qPCR). Data are presented as mean ± SD of three independent animal series. WT: black line, *Usp2*-KO: grey line.

**Fig 6:**
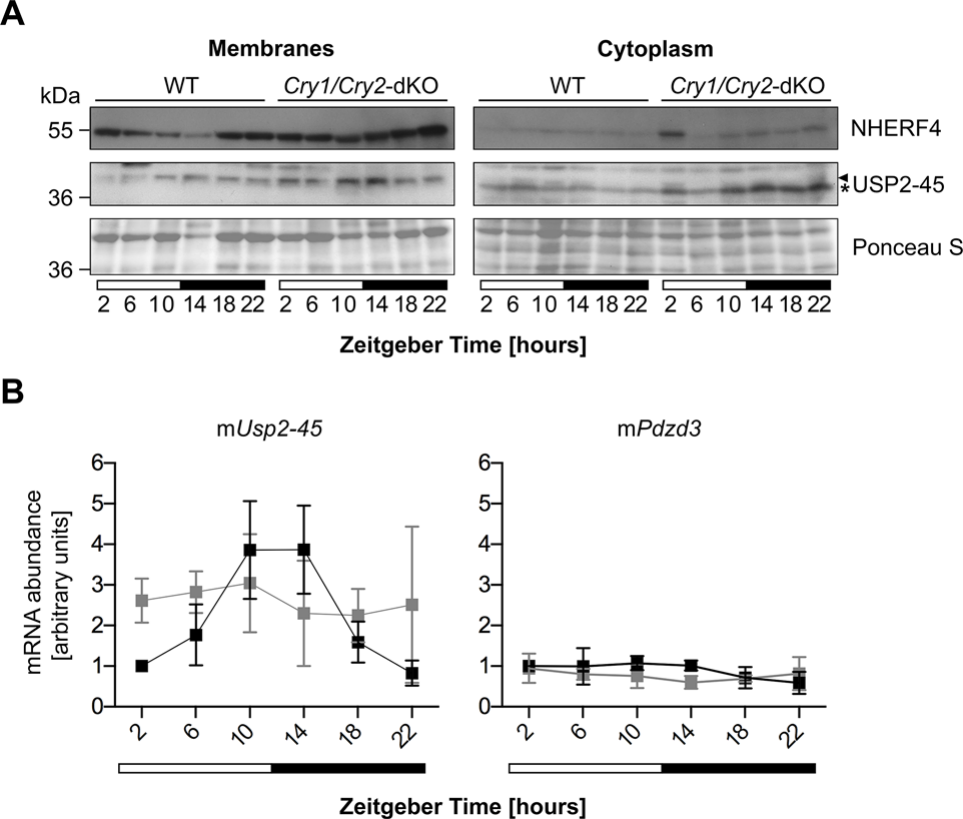
USP2-45 and NHERF4 display lower amplitude rhythms in *Cry1/Cry2*-dKO mice. **A**: Mucosa of the whole small intestine was dissected at the indicated time points around the clock and membrane and cytoplasmic protein extracts were prepared from the remaining tissue. Protein extracts were analysed by immunoblotting with anti-USP2-45 N-term (arrow: USP2-45, *: non-specific) and anti-NHERF4 antibodies. Ponceau-Red S staining of the membrane was used as loading control. Data are western blots from one representative series of mice out of two. **B**: RNA was extracted from mid-jejunum and the mRNA levels of the corresponding proteins were analysed by semi-quantitative real-time PCR (qPCR). Data are presented as mean ± SD of three independent animal series. WT: black line, *Cry1/Cry2*-dKO: grey line.

We subsequently compared membrane and cytoplasmic fractions of small intestine *in vivo* by biochemical separation [45] and observed a constant and strong up-regulation of NHERF4 at all time-points taken around the clock in membranes fractions of *Usp2*-KO compared to WT littermates (Fig 5A). This phenomenon occurs at the protein level since the expression of the *Pdzd3* mRNA encoding NHERF4 is not affected in *Usp2*-KO (Fig 5B). As expected from our previous data in the kidney [25], USP2-45 is also rhythmically expressed in the small intestine in a phase that closely follows its mRNA and was found in the membranes fraction. In WT mice, NHERF4 abundance appears to be antiphasic to USP2-45, suggesting that it is negatively regulated at the protein level (Fig 5A, S6 Fig). We took advantage of the disturbed expression of USP2-45 existing in *Cry1/Cry2*-dKO mice, which lack a functional clock [46, 47], to further study the action of USP2-45 on NHERF4. Despite different genetic backgrounds (*Usp2*-KO: C57BL6/N, *Cry1/Cry2*-dKO: C57BL6/J), we observed similar expression patterns of mRNA and protein expression for both USP2-45 and NHERF4 in control mice of both lines (Figs 5B and 6B, S7 Fig). However, in contrast to data in the liver [3], *Usp2-45* mRNA is not overexpressed in the small intestine of *Cry1/Cry2*-dKO, but displays a lower amplitude rhythm around the clock. In turn, NHERF4 is not down or up-regulated but keeps a lower amplitude post-transcriptional rhythm (Fig 6A). These data indicate that the levels of NHERF4 have an inverse relation to the ones of USP2-45, suggesting that USP2-45 negatively regulates NHERF4 at the protein level.

## Discussion

Up-to date, the suspected role of USP2 in the post-translational regulation of core clock components remains unclear [24, 27]. Since the impact of *Usp2* deletion gives at best minor effects on circadian behavior, the distinction between a modest involvement of *Usp2* or a functional compensation by one of the numerous existing DUBs [18] remains a challenge. In the present study, we overcame this difficulty by using both mouse and *Drosophila* models of *Usp2* knockdown, assuming that its core clock cogwheel or output effector status may be conserved throughout evolution. We confirmed that CG14619, which is a CCG [37, 38], is the orthologue of m*Usp2* by protein sequence alignments (S1 Fig) and that d*Usp2*-kd suffer from sub viability ([33]; Fig 2C–2F, S1 and S2 Files). Interestingly, d*Usp2* and d*Usp8*, which is a core clock cogwheel in *Drosophila* are paralogues and possibly differentially evolved towards output effector and core cogwheel status, respectively [36, 48]. We found no significant alteration of the circadian free-running period in both clock-neuron specific knockdown of d*Usp2* in *Drosophila* and in our *Usp2*-KO mouse (Fig 1, Fig 2A and 2B, Table 1). In addition, acute silencing of *USP2* in human osteosarcoma U2-OS cells does not lead to an obvious alteration of the free-running period measured by bioluminescence [29]. The RNAi construct we used yields viable flies without affecting off-target genes as it is stated in the data of the Vienna Drosophila Resource Center (VDRC v104382 detailed view). Given the phenotype observed in whole-body knockdown, we assumed the knockdown efficiency may be comparable in the clock neurons. This has to be taken into account in the conclusion made above. In addition our data are partly conflicting with the previous studies on the circadian role of *Usp2*. Indeed, similarly to Besharse and coworkers, we do not observe an increase in free-running period reported by Cermakian and coworkers. However, our *Usp2*-KO mice do not display an increased locomotor activity. Taken altogether, these data indicate a potentially compensated role in the central clock.

Having established this framework, we investigated the hypercalciuric phenotype of *Usp2*-KO mice by using dietary Ca^2+^ restriction, fasting, analyses of bone structure by micro-computed tomography, measures of circulating level of PTH and Vitamin D and expression level of their respective target genes. We could clearly establish from these data that the observed hypercalciuria essentially results from intestinal hyperabsorption rather than from other causes, namely enhanced bone resorption, renal leak or endocrine defects in calciotropic hormones (Fig 3A–3C, S2 and S3 Figs); For a review, see [31]). The bodily balance of Ca^2+^ relies on dynamic fluxes between the tissues involved in its homeostasis in order to maintain plasma Ca^2+^ within its physiological range. In the present situation, *Usp2*-KO mice live in a steady-state in which an overall increased intake of Ca^2+^ results in an increased excretion. Furthermore, the phenotype is likely caused by the lack of USP2-45, which is the only detectable USP2 isoform in mouse intestine ([16, 43]; S4 Fig). In terms of human medicine, disruption of circadian rhythms represents a major risk for nephrolithiasis, the formation of kidney stones (reviewed in [49]). This phenomenon may result from impaired rhythm in the expression of hUSP2-45. Indeed, it was recently reported that 14 monogenic genes account for 15% of nephrolithiasis [50]. This percentage may increase by screening nephrolithiasis patients for mutations in *USP2* or in clock genes controlling its expression such as *ARNTL* (*BMAL1*) and *CLOCK* and their binding sites in the promoter of USP2. This interesting perspective is supported by the conservation of *Usp2* towards bodily Ca^2+^ homeostasis, which is evidenced by the Ca^2+^-dependent rescue of d*Usp2*-kd flies (Fig 3). The relationship between the gene and the physiological process nevertheless appears to be antagonist between mammalian and insect experimental models. This fact could however be explainable by the possible divergent evolution of molecular cascades, number and nature of putative intermediates, which may have taken place since the two species shared their last common ancestor.

Based on these findings, we therefore pursued our analysis of the role of USP2-45 as clock output effector in mouse small intestine. Quantitative proteomics allowed us to identify the membrane scaffolding protein NHERF4 as one of the very few deregulated proteins in *Usp2*-KO out of a detectable proteome of 3590 (S5 Fig), making NHERF4 a candidate target of USP2-45. Accordingly, USP2-45 interacts with NHERF4 and endogenous Clathrin Heavy Chain (CLH) in transfected HEK293 cells (Fig 4). This suggests that the three proteins are possibly part of a complex at the cell membrane, which is not the case with the non-circadian isoform USP2-69. The molecular determinants conferring these properties to USP2-45 likely reside in its 49 N-terminal amino acids since both isoforms share the same catalytic domain and have different substrate specificities [16, 21]. Our present finding is however in opposition with previous data from the Loda lab indicating that hUSP2-69 interacts with Clathrin Heavy Chain [51, 52]. However, these data were obtained by affinity chromatography in human LNCaP cells, thus experimental, species and/or tissue specificities may explain these discrepancies. Finally, in *Schizosaccharomyces pombe*, an organism in which most DUBs are found in complexes, UBP4 is the closest relative to mUSP2-45 according to pBLAST and is associated with SFP47, an SH3-domain containing protein acting in endocytosis [53]. These data suggest a conserved role in processes related to regulation of membrane proteins among mUSP2-45 and its ancestors.

We subsequently analyzed the expression pattern of NHERF4 together with that of USP2-45 in membrane and cytoplasmic extracts of small intestine of WT, *Usp2*-KO and *Cry1/Cry2*-dKO and report three important findings. First, accordingly to our *in vitro* data, USP2-45 was found in the membranes fraction in the intestinal epithelium, although previous studies located it in the nucleus [20] or the peroxisomes [23]. Second, in WT animals, NHERF4 presents a rhythmic expression in anti-phase to USP2-45 at the protein level, since the levels of its mRNA is not rhythmic and unaltered in both *Usp2*-KO and *Cry1/Cry2*-dKO (Figs 5 and 6). Third, the deregulation of USP2-45 expression in these mice models allowed us to establish the inverse relationship between USP2-45 and NHERF4 (i.e. rhythmic in WT, constant increased level in *Usp2*-KO and medium but constant level in *Cry1/Cry2*-dKO). As mentioned above, NHERF4 regulates the activity of TRPV6, the main Ca^2+^ channel involved in dietary Ca^2+^ uptake [32]. This latter phenomenon is rhythmic in rats [54–56] and the post-translational regulation of NHERF4 we report might be partly responsible for this observation. The lack of a deubiquitylating enzyme leading to the stabilization of a target protein is not common with respect to the most known function of such enzymes towards proteasomal degradation-promoting signals such as Lys48-linked polyubiquitin chains amongst others [57]. This idea is supported by previous data of our laboratory suggesting that USP2-45 can promote the degradation of the mineralocorticoid receptor upon stimulation with its ligand aldosterone [58]. In turn, between its translation and its scaffolding function at the membrane, NHERF4 may likely be post-translationally modified with non-degradative Ubiquitin, or Ubiquitin-like modifiers such as SUMO, NEDD8 or ISG15, which can all be removed by USP2 [18, 19]. Such non-degradative signals could regulate its cellular transport, its stability, or its association with its target proteins, a view that is supported by the presence of 2 ubiquitylation sites in the PDZ domains of mouse NHERF4 [59]. Very interestingly, the *Pdzd3* gene encoding NHERF4 is only 152 kb away and the fourth protein-coding gene downstream of *Usp2* on mouse chromosome 9 (*Usp2*: 44’067’021 to 44’095’627 bp; *Pdzd3*: 44’247’312 to 44’251’464 bp). This genomic proximity is conserved amongst vertebrates from *Xenopus laevis* to *Homo sapiens* and is in line with a common function, as suggested by gene clustering studies transposing the concept prokaryotic operons to eukaryotic genomes [60, 61].

**Fig 7:**
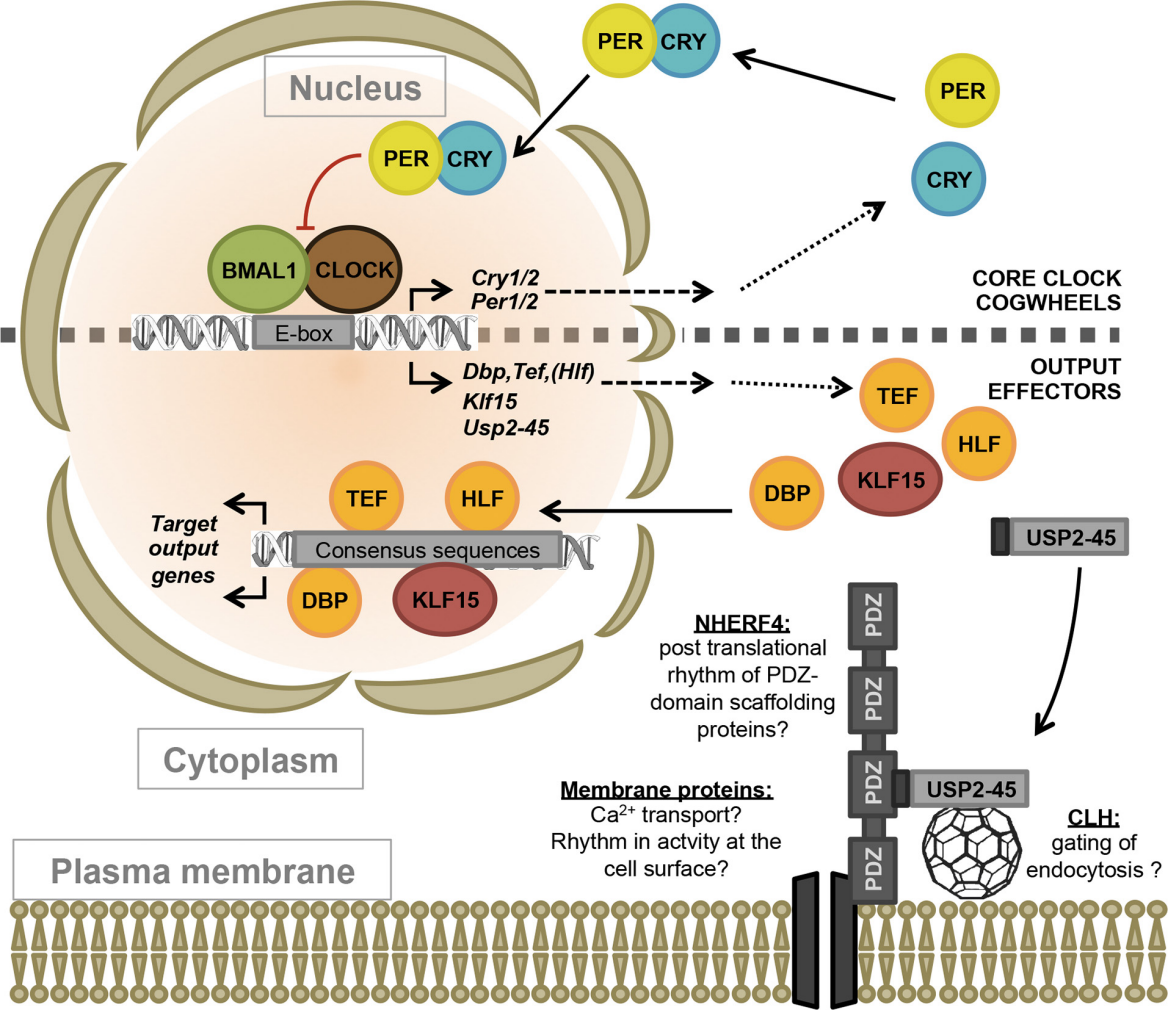
Proposed model of body-wide clock output effectors. Based on the data presented above, USP2-45 becomes the first non-transcriptional clock output effector along with DBP, TEF, HLF and KLF15. Given the biochemical location of USP2-45 in membranes and its action on NHERF4 *in vivo* and its interaction with NHERF4 and the ubiquitous CLH *in vitro*, we propose that USP2-45 may act similarly in other tissues. Possible role could be related to Ca^2+^ transport across the cell membrane, indirect rhythmic regulation of membrane protein function by PDZ-domain containing scaffolds or circadian gating of cell sensitivity to circulating cues by rhythmic endocytosis.

Our data lead us to propose a new model of the circadian clockwork placing USP2-45 along with DBP, HLF, TEF and KLF15 as an output effector acting at the protein level, preferentially at the cell surface and possibly to regulate membrane permeability of Ca^2+^ (Fig 7). Total and constitutive KO mouse models may only partially reveal the function of a gene through observable phenotypes. However, several published data offer exciting perspectives on the role of USP2-45 in regulation of Ca^2+^-dependent processes. First, the other important phenotype reported in *Usp2*-KO mice is a severe male subfertility. *Usp2*-KO spermatozoa are immotile in PBS, but not in M199 medium containing Ca^2+^, amongst other solutes [30]. Given that sperm motility and acrosome reaction are highly dependent on Ca^2+^[62], it is possible that the male subfertility reported by Wing and colleagues relies on disturbed Ca^2+^ homeostasis in *Usp2*-KO spermatozoa. A recent study suggests a physiological role in regulation of CaV_1.2_ Ca^2+^ channels in the heart [63]. Finally, oscillations in intracellular Ca^2+^ are of importance in circadian biology in various cell types across kingdoms [64–67] and USP2-45 may participate in a rhythmic regulation of intracellular Ca^2+^ by controlling plasma or intracellular stores membranes permeability of Ca^2+^. Further studies based on isoform, tissue-specific and/or inducible *Usp2-45*-knockout mouse or cell models are however needed to further investigate *in vivo* the role of USP2-45. Altogether, these findings raise exciting perspectives under the new paradigm of USP2-45 as a clock-output membrane effector and its potential role in rhythmic control of membrane proteins dynamics and membrane permeability to Ca^2+^.

## Materials and Methods

### Mouse models

*Usp2*-KO mice were bred as previously described [25]. *Cry1/Cry2*-dKO mice with C57BL6/J genetic background [46, 47] were bred as described in [68]. All experimental procedures were approved by the Swiss animal welfare authorities (Office of Veterinary Affairs of the Canton de Vaud, Switzerland) and carried out in accordance with the local animal welfare act. Mice were sacrificed as indicated in the corresponding experimental procedures sections by decapitation or by cervical dislocation after irreversible anesthesia (0.8 mg Xylazine and 1 mg Ketamine per kg of body weight in 0.9% NaCl injected intraperitoneally).

*Running wheels*. 8-week old *Usp2*-KO males and WT littermates were housed in light tight boxes and habituated for 2 weeks to 12h Light:12h Dark cycles (LD) in individual cages equipped with a running wheel (Coulbourn Instruments). Locomotor activity was measured for 1 week in LD and the mice were released in constant darkness (DD) for 3 weeks. Free running activities were calculated by using Fourier’s transformation with the Matlab software (Clocklab).

### *Drosophila* circadian and locomotor activity

Knockdown of CG14619 in *Drosophila* was achieved by using the UAS-Gal4 system. Flies homozygously carrying the UAS-RNAi construct directed against all the transcripts of CG14619 (CG14619^KK108078^) were obtained from the Vienna Drosophila Resource Center (clone v104382) and crossed with *tub-Gal4* / *TM3*,*Sb* (Bloomington Stock Centre no. 5138) and *tim-Gal4* / *CyO* [69] GAL4 driver lines. Flies were kept at 25°C and entrained for 3 days in a 12h:12h LD cycle, followed by constant darkness for 10 days. Locomotor activity was recorded using Drosophila Activity Monitors (Trikinetics Inc, USA) and the data analysed using FaasX software [70]. Actograms were double-plotted with 30 min resolution and hash density 12. Statistical tests were performed using GraphPad Prism software (GraphPad Software, USA).

### *Drosophila* survival studies

Standard fly food was enriched as follows: Food volume in vial was measured and food was quickly melted in a microwave oven. For the CaCl_2_ dose response experiment, the appropriate volume of warmed 30 mM, 100 mM, 300 mM and 1M CaCl_2_ was added to obtain the final concentrations of 1, 3, 10 and 30 mM CaCl_2_, respectively. For the Ca^2+^-specificity experiment 1M CaCl_2_, 1M MgCl_2_, 1M NaCl, 2M NaCl yielded the final concentrations of 30 mM CaCl_2_, 30 mM MgCl_2_, 30 mM NaCl, 60 mM NaCl, respectively. H_2_O was similarly used as a negative control and mixture was homogenized by pipetting, stored at 4°C and enriched with penicillin/streptomycin. The homozygous CG14619^KK108078^ UAS-RNAi line was crossed at 25°C with the tubulin-Gal4 driver line balanced over the *TM6b* balancer carrying the "tubby" dominant marker permitting selection of the tubulin-Gal4-carrying progeny from the control siblings. Pupal cases were counted after pupation of the latest wandering 3^rd^ instar larvae. Adults were daily sorted after hatching and Mendelian proportions were calculated.

### Metabolic cages

8–12 week old *Usp2*-KO and WT mice were fed a standard housing chow (Kliba-Nafag) and were habituated to LD for at least 2 weeks. The animals were placed in individual metabolic cages (Techniplast, Buguggiate, Italy) for body weight, water and food consumption measurements and urine collection every 4 hours for 32 hours in LD. The data from overlapping ZT intervals were pooled. For the fasting experiment, the animals were studied for two consecutive days starting at ZT12 (light offset, end of resting phase) and fed a standard housing chow on the first day and fasted from ZT12 on the second. Urine samples were collected at the end of the ZT12-18, ZT18-09 and ZT09-12 time intervals.

### Plasma and urine analyses

Plasma Ca^2+^, urine Ca^2+^and creatinine were measured by the clinical chemistry lab of the Lausanne University Hospital (CHUV-LCC) by using the O-cresolphtalein complexone and Jaffe reaction, respectively. Total plasma Ca^2+^ was calculated with the Parfitt’s [71] calculation of albumin-bound Ca^2+^:

$${\left[{Ca}^{2+}\right]}_{\text{corr.}}={\left[{Ca}^{2+}\right]}_{\text{total}}-0.012\times \frac{\left[Albumin\right]}{0.9677-39.55}$$

### Protein extraction and immunoblotting

*Usp2*-KO, *Cry1/Cry2*-dKO and WT male littermates or C57BL6/J controls were entrained for 3 weeks to normal or inverted LD cycles in light tight housing boxes (Coulbourn Instruments, Whitehall, USA) and fed a standard housing chow (Kliba-Nafag #3800, Kaiseraugst, Switzerland). The animals were sacrificed by decapitation under white or red dim light and the whole small intestine was isolated and rinsed in ice cold HBSS containing protease inhibitor cocktail (Roche) and 0.5 mM PMSF. Membrane and cytoplasmic fractions were obtained by using the technique of Biber for preparation of Brush Border Membrane Vesicles (BBMV) [45]. However, purity checking revealed significant presence of basolateral and ER membranes. We therefore named the fractions membranes instead of BBMV. 30 μg of extract were separated on SDS-PAGE and transferred onto PVDF membranes. The following antibodies were used at the indicated dilutions: anti-USP2-45-Nterm and anti-USP2-Cterm (1/50; [25]), anti-NHERF4 (1/5000; LifeSpan Biosciences, Inc. Seattle, USA), anti-CLH TD.1 (1/200; Santa Cruz), anti-βACTIN (1/1000; Sigma-Aldrich, St-Louis, USA), anti-βTUBULIN (1/1000; Sigma-Aldrich, St-Louis, USA). Signal was revealed by chemiluminescence ECL reagent (Amersham) and detected on autoradiography films (Amersham). Quantification of western blots were achieved by densitometry, Ponceau S was used as a loading control and data were normalized to WT at ZT 2.

### Affinity precipitation

HEK293 cells were seeded in 100mm dishes and transiently transfected with the following plasmids: empty pCDNA3.1 (10 μg), S-tagged mUSP2-45 (5 μg), S-tagged mUSP2-69 (10 μg) and pCMV-SPORT6-hNHERF4 (Open Biosystems) using lipofectamine 2000 (Invitrogen). 24 hours after transfection cells were lysed and precipitation was performed at 4°C as described previously [72] and scaled to 200 μg protein input.

### Semi-quantitative RT-PCR

Total RNA was extracted from small intestinal mucosa or one half of the left kidney using a standard Phenol / Chloroform method. Two μg of total RNA was retro-transcribed as described previously [25] using Superscript II retro-transcriptase (Invitrogen). Real time quantitative PCR was performed using a Roche 480 Light Cycler system on cDNA corresponding to 20 ng of initial RNA by using the following TaqMan® (Applied Biosystems) gene expression assays: *Usp2-45* [73], *Pdzd3*: Mm00466964_g1, *Cyp24a1*: Mm00487244_m1; *S100g*: Mm00486654_m1; *Gapdh*: Mm99999915_g1. mRNA relative abundance was calculated using the standard regression curve method and normalized to *Gapdh*.

### Statistical analyses

Outliers were removed from datasets using the 1.5 interquartile range (1.5-IQR) exclusion criterion and the presented data are mean ± SD when individual values were not plotted. Multiple comparisons were performed by two-way ANOVA and post-hoc two-tailed Student T-tests were performed using the Holm-Sidak correction of significance threshold for multiple testing if not stated otherwise.

## Supporting Information

S1 Fig CG14619 is the orthologue of mUSP2. The sequences of the 4 reported mammalian orthologues of dCG14619 were aligned and the conserved domains of Ubiquitin-specific proteases were compared. These domains comprise a stretch of amino acids around the catalytic cysteine residue (Cys Box, **A**), the glutamine-aspartate-glutamate triad (QDE Box, **B**) and the conserved histidine (His Box, **C**). **D**: The percentage of homology in these three domains between dCG14619 to its four mammalian orthologues was calculated and is reported.
(TIF)

S2 Fig *Usp2*-KO mice do not develop hypocalcemia on dietary Ca^2+^ restriction and have unaltered excretion of Mg^2+^ and PO43-. **A, B**: Plasma was collected at ZT13 after 50 days (**A**) and 6 months (**B**) of dietary Ca^2+^ restriction. Ca^2+^ concentration was calculated using the Parfitt’s correction for albumin-bound fraction. **C, D**: Urine from mice placed in individual metabolic cages was collected during the indicated time intervals. The molar ratio of Mg^2+^ (**C**) and PO_4_^3-^ (**D**) to creatinine was calculated and plotted as individual values of 12–18 samples obtained from 12 animals per genotype. Black dots: WT, Gray dots: *Usp2*-KO, bar = median.
(TIF)

S3 Fig *Usp2*-KO have no disturbances in calciotropic hormones circulating levels. **A, B**: PTH concentration was assayed by ELISA in plasma collected on mice fed a standard housing chow (**A**) or maintained under long-term dietary Ca^2+^ restriction (**B**) at the acrophase of USP2-45 expression (ZT13). C: Expression levels of PTH target genes in the kidney were found in previously obtained renal transcriptome analysis dataset (NCBI-GEO accession: GSE43517). **D-F**: 25-(OH)-D (D) and 1,25-(OH)_2_-D (**E, F**) concentrations were assayed by radioimmunoassay. **G, H**: *Cyp24a1* and *S100g* expression levels in *Usp2*-KO and WT littermates were measured by semi-quantitative RT-PCR in kidney and duodenum, respectively. N = 12 (**G**), n = 6 (**H**). Data are presented as individual values. Black dots: WT; Gray dots: *Usp2*-KO. n = 14–16 (**A**), 5–7 (**B**), 6–10 (**D, E**) and 4–6 (**F**) animals per genotype.
(TIF)

S4 Fig USP2-45 is the only detectable USP2 isoform in the small intestine. Total proteins were extracted from skeletal muscle (**A**), Heart (**B**) and small intestine (**C**) and analysed by immunoblotting with N and C-terminal anti-USP2 antibody for USP2-45 and USP2-69 detection, respectively.
(TIF)

S5 Fig Quantitative proteomics identified NHERF4 as a strongly up-regulated protein in *Usp2*-KO duodenal mucosa. Proteins quantified by iTRAQ 8-plex are presented in a volcano plot. X-axis shows, for each protein, logarithmized ratio of the median of normalized iTRAQ intensities for KO channels divided by the median of normalized iTRAQ intensities for WT channels. Y-axis shows the negative logarithm of the p-value obtained from Local-Pooled-Error testing. 5 proteins (shown in red) on a total of 3590 are statistically considered as significantly differentially expressed (False Discovery Rate < 0.05).
(TIF)

S6 Fig The lack of USP2-45 leads to a loss of NHERF4 rhythm at the protein level in the membrane compartments of small intestine mucosa. Western-blotting data shown on Fig 5 were quantified by densitometry. Data are presented as mean ± Standard Error of the Mean (SEM) of 2 independent experiments.
(TIF)

S7 Fig USP2-45 and NHERF4 display lower amplitude rhythms in *Cry1/Cry2*-dKO mice. Western-blotting data shown in Fig 5 were quantified by densitometry. Data are presented as mean ± Standard Error of the Mean (SEM) of 2 independent experiments.
(TIF)

S1 File Actogram of control flies (tub>Gal4/+). (PDF)

S2 File Actogram of Usp2 knockdown flies (tub>UAS d*Usp2*^KK108078^ RNAi). (PDF)

S1 Table *Usp2*-KO mice fed a standard diet for 12 months do not develop osteoporosis. The femora of 9 *Usp2*-KO and 9 WT littermates were analysed by micro computed tomography (micro CT). Abbreviations: Full bone parameters (FULL): AVD: Apparent Volume Density; Cortical bone parameters (CORT): %BV: Cortical Bone Volume Density, Ct.Th: Cortical Thickness, J, Imax, Imin: Polar Moments of Inertia; Trabecular bone parameters (TRAB): BV/TV: Trabecular Bone Volume Density, BS/TV: Trabecular Bone Surface Density, BS/BV: Specific Bone Surface, Tb.Th: Trabecular Thickness. Tb.Sp: Trabecular Separation, Tb.N: Trabecular Number, Conn.D: Trabecular Connectivity Density. *: p<0.01
(PDF)

S2 Table *Usp2*-KO mice fed a 0.02% low Ca^2+^ diet for 6 months maintain normal bone structure. The femora of 4 *Usp2*-KO and 6 WT littermates were analysed by micro computed tomography (micro CT). Abbreviations: Full bone parameters (FULL): AVD: Apparent Volume Density; Cortical bone parameters (CORT): %BV: Cortical Bone Volume Density, Ct.Th: Cortical Thickness, J, Imax, Imin: Polar Moments of Inertia; Trabecular bone parameters (TRAB): BV/TV: Trabecular Bone Volume Density, BS/TV: Trabecular Bone Surface Density, BS/BV: Specific Bone Surface, Tb.Th: Trabecular Thickness. Tb.Sp: Trabecular Separation, Tb.N: Trabecular Number, Conn.D: Trabecular Connectivity Density.
(PDF)

S1 Text Supplementary experimental procedures. (DOCX)
