# Supplementary figures and images for: USP2-45 Is a Circadian Clock Output Effector Regulating Calcium Absorption at the Post-Translational Level
Source: PLoS One. 2016 Jan 12;11(1):e0145155. doi: 10.1371/journal.pone.0145155 (PMC4710524; doi:10.1371/journal.pone.0145155)

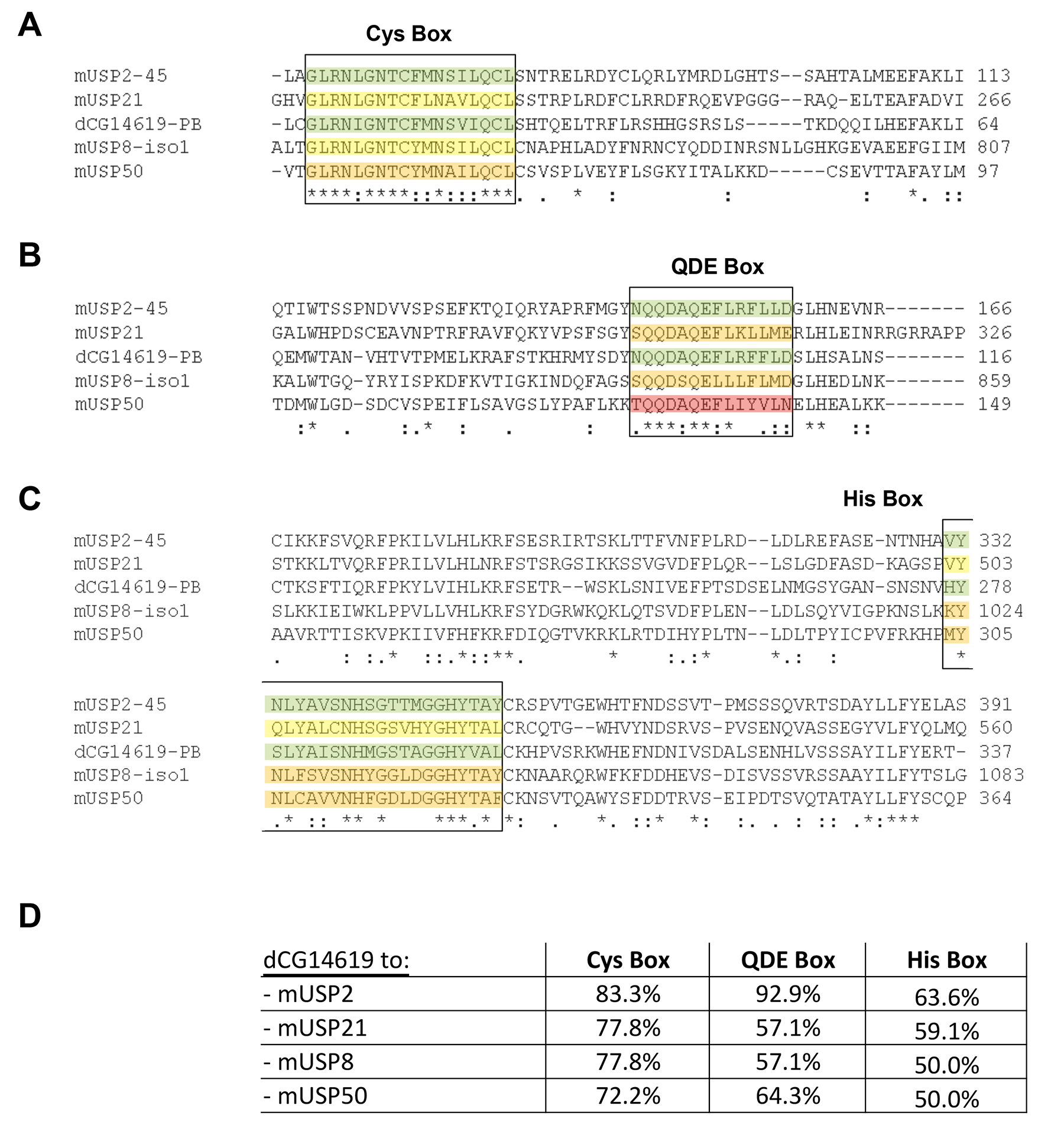

Supplement: S1 Fig — The sequences of the 4 reported mammalian orthologues of dCG14619 were aligned and the conserved domains of Ubiquitin-specific proteases were compared. These domains comprise a stretch of amino acids around the catalytic cysteine residue (Cys Box, A), the glutamine-aspartate-glutamate triad (QDE Box, B) and the conserved histidine (His Box, C). D: The percentage of homology in these three domains between dCG14619 to its four mammalian orthologues was calculated and is reported. (TIF) [file pone.0145155.s001.tif]

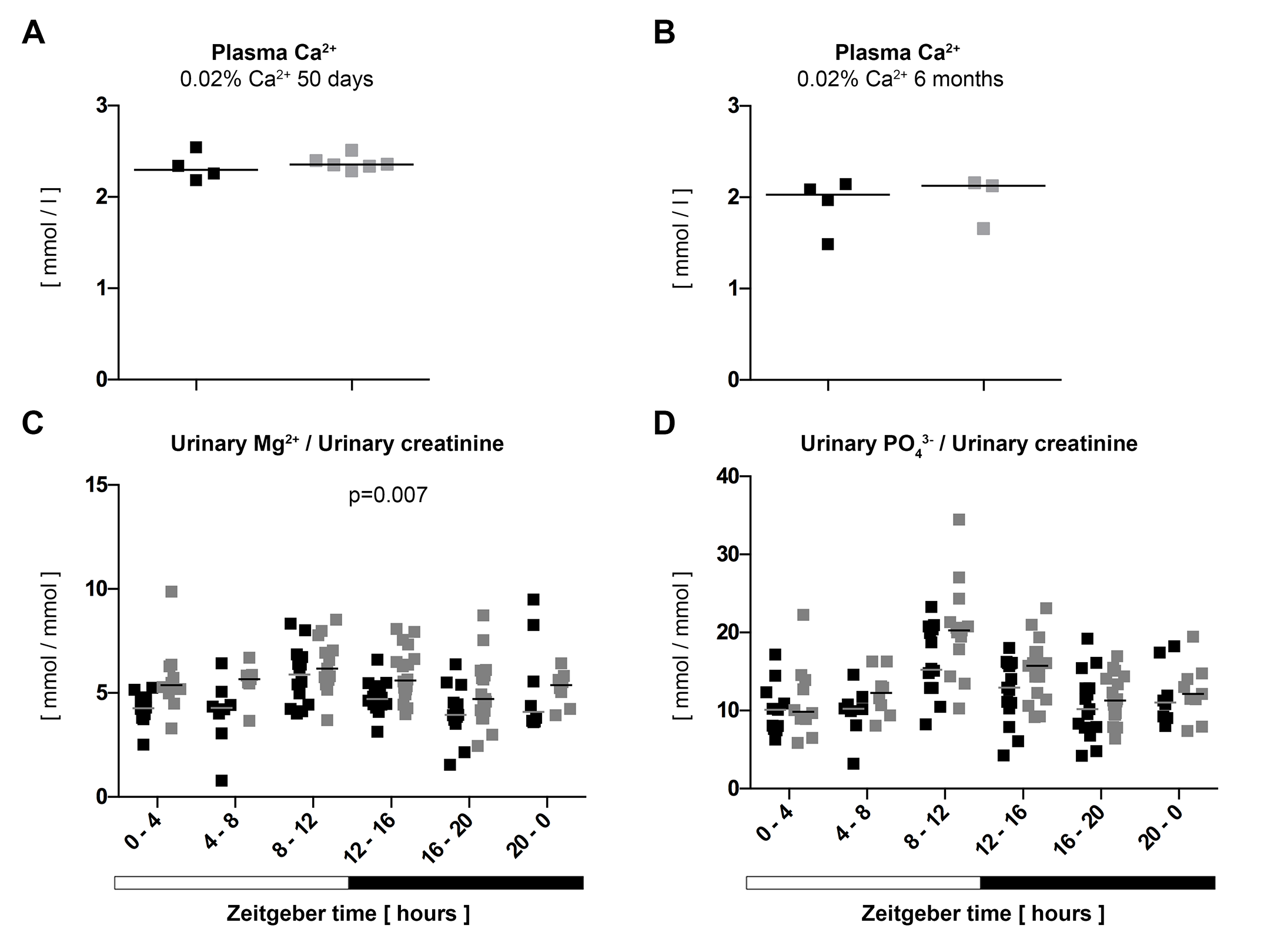

Supplement: S2 Fig — A, B: Plasma was collected at ZT13 after 50 days (A) and 6 months (B) of dietary Ca2+ restriction. Ca2+ concentration was calculated using the Parfitt’s correction for albumin-bound fraction. C, D: Urine from mice placed in individual metabolic cages was collected during the indicated time intervals. The molar ratio of Mg2+ (C) and PO43- (D) to creatinine was calculated and plotted as individual values of 12–18 samples obtained from 12 animals per genotype. Black dots: WT, Gray dots: Usp2-KO, bar = median. (TIF) [file pone.0145155.s002.tif]

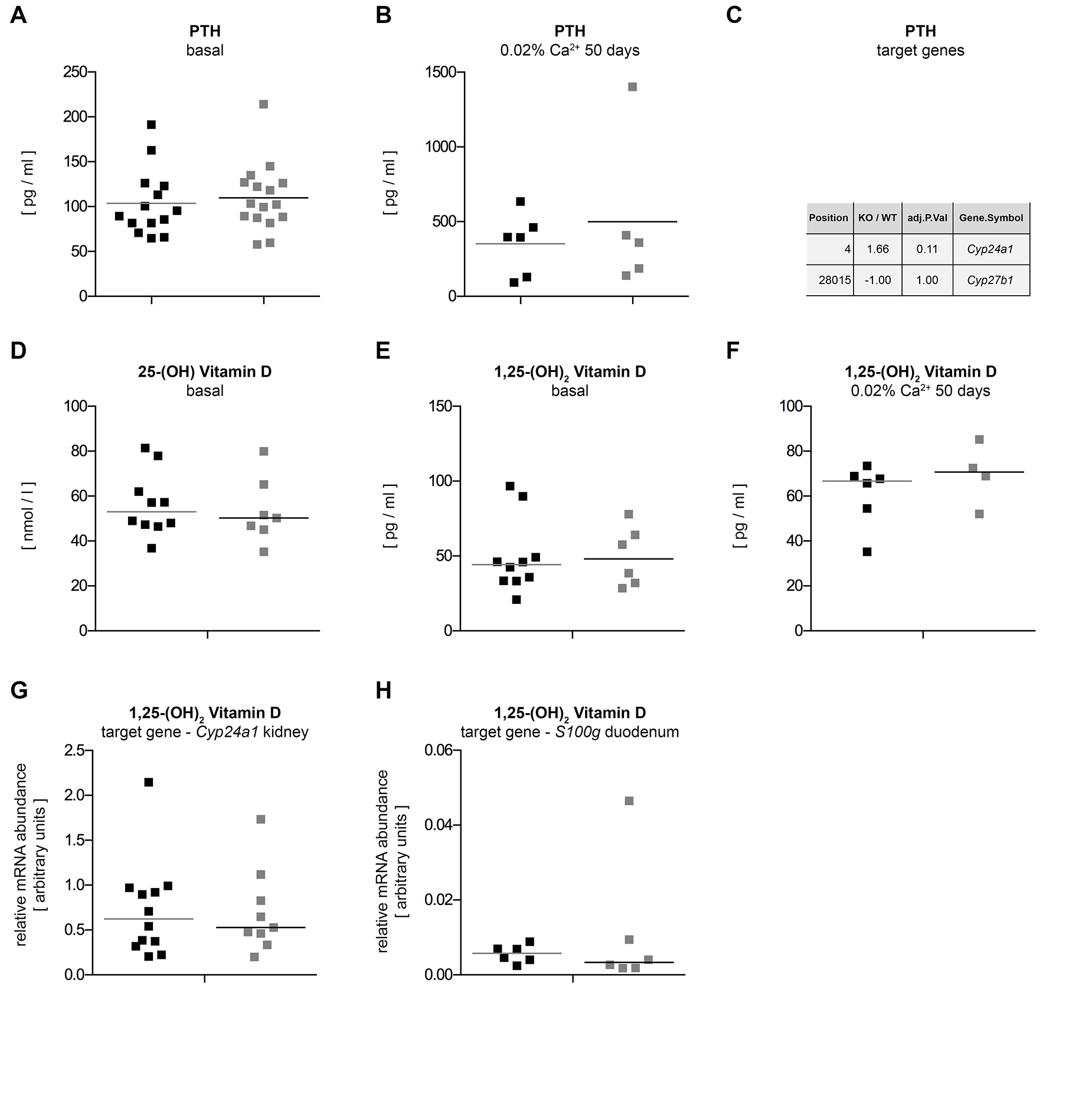

Supplement: S3 Fig — A, B: PTH concentration was assayed by ELISA in plasma collected on mice fed a standard housing chow (A) or maintained under long-term dietary Ca2+ restriction (B) at the acrophase of USP2-45 expression (ZT13). C: Expression levels of PTH target genes in the kidney were found in previously obtained renal transcriptome analysis dataset (NCBI-GEO accession: GSE43517). D-F: 25-(OH)-D (D) and 1,25-(OH)2-D (E, F) concentrations were assayed by radioimmunoassay. G, H: Cyp24a1 and S100g expression levels in Usp2-KO and WT littermates were measured by semi-quantitative RT-PCR in kidney and duodenum, respectively. N = 12 (G), n = 6 (H). Data are presented as individual values. Black dots: WT; Gray dots: Usp2-KO. n = 14–16 (A), 5–7 (B), 6–10 (D, E) and 4–6 (F) animals per genotype. (TIF) [file pone.0145155.s003.tif]

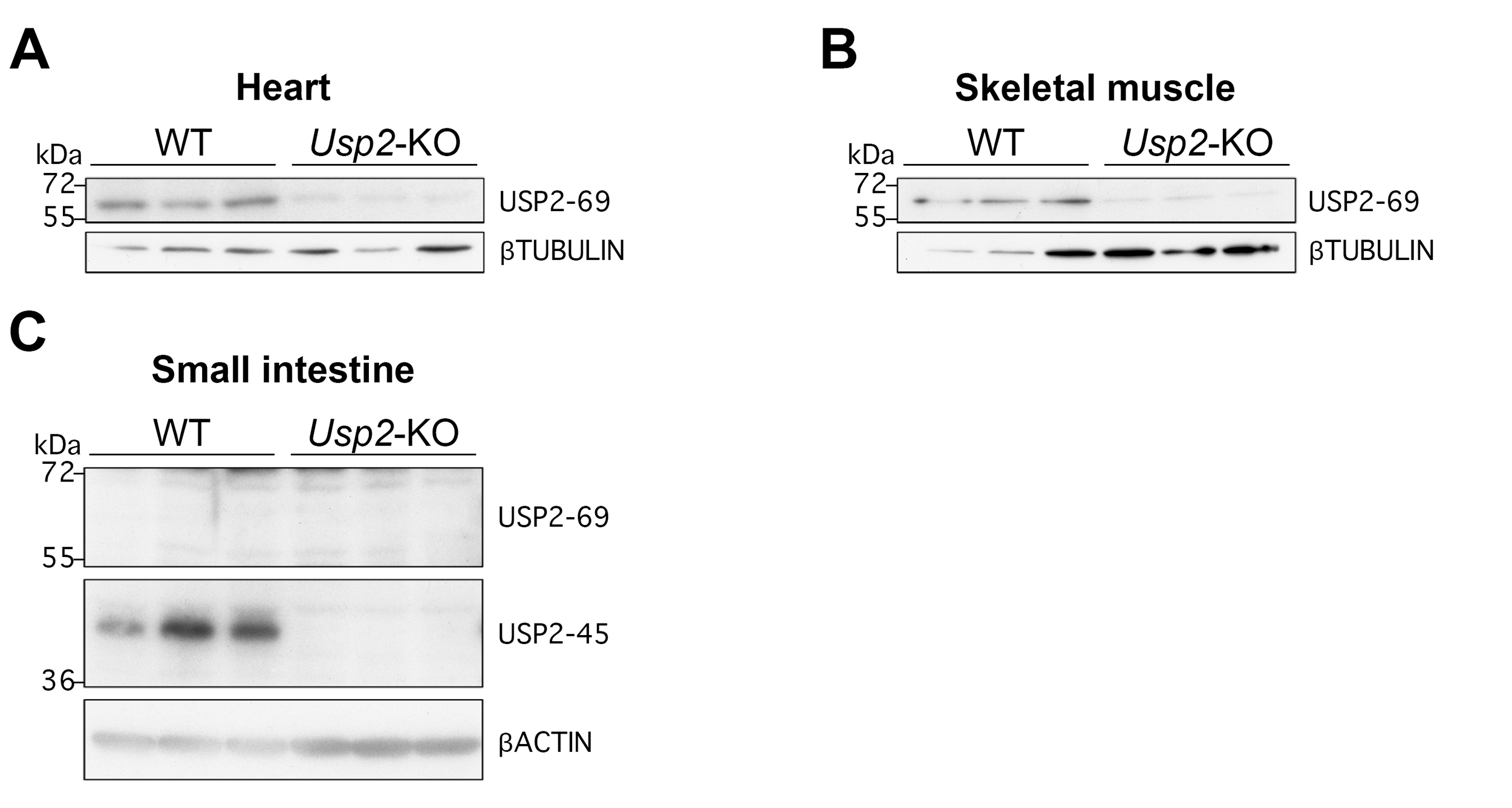

Supplement: S4 Fig — Total proteins were extracted from skeletal muscle (A), Heart (B) and small intestine (C) and analysed by immunoblotting with N and C-terminal anti-USP2 antibody for USP2-45 and USP2-69 detection, respectively. (TIF) [file pone.0145155.s004.tif]

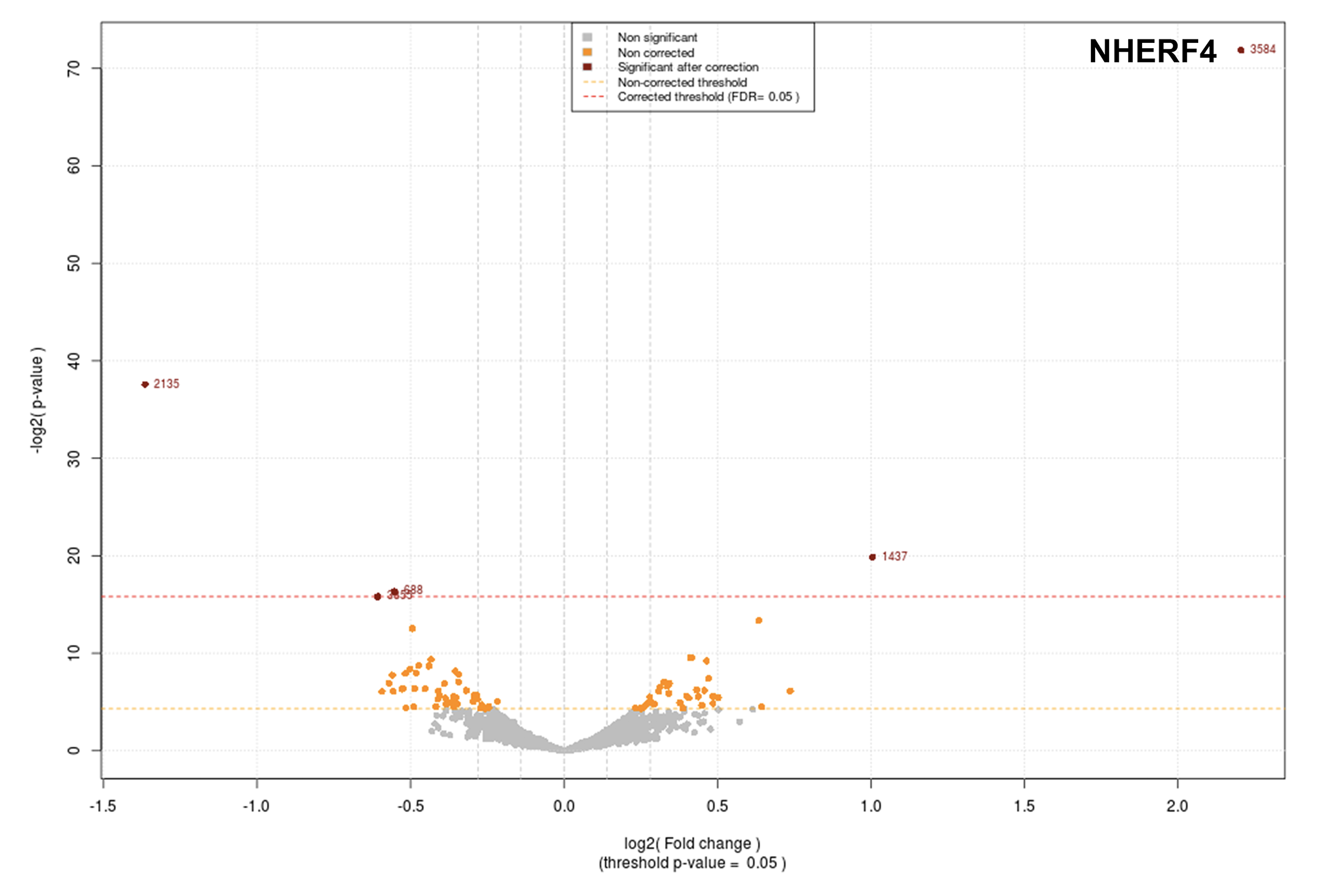

Supplement: S5 Fig — Proteins quantified by iTRAQ 8-plex are presented in a volcano plot. X-axis shows, for each protein, logarithmized ratio of the median of normalized iTRAQ intensities for KO channels divided by the median of normalized iTRAQ intensities for WT channels. Y-axis shows the negative logarithm of the p-value obtained from Local-Pooled-Error testing. 5 proteins (shown in red) on a total of 3590 are statistically considered as significantly differentially expressed (False Discovery Rate < 0.05). (TIF) [file pone.0145155.s005.tif]

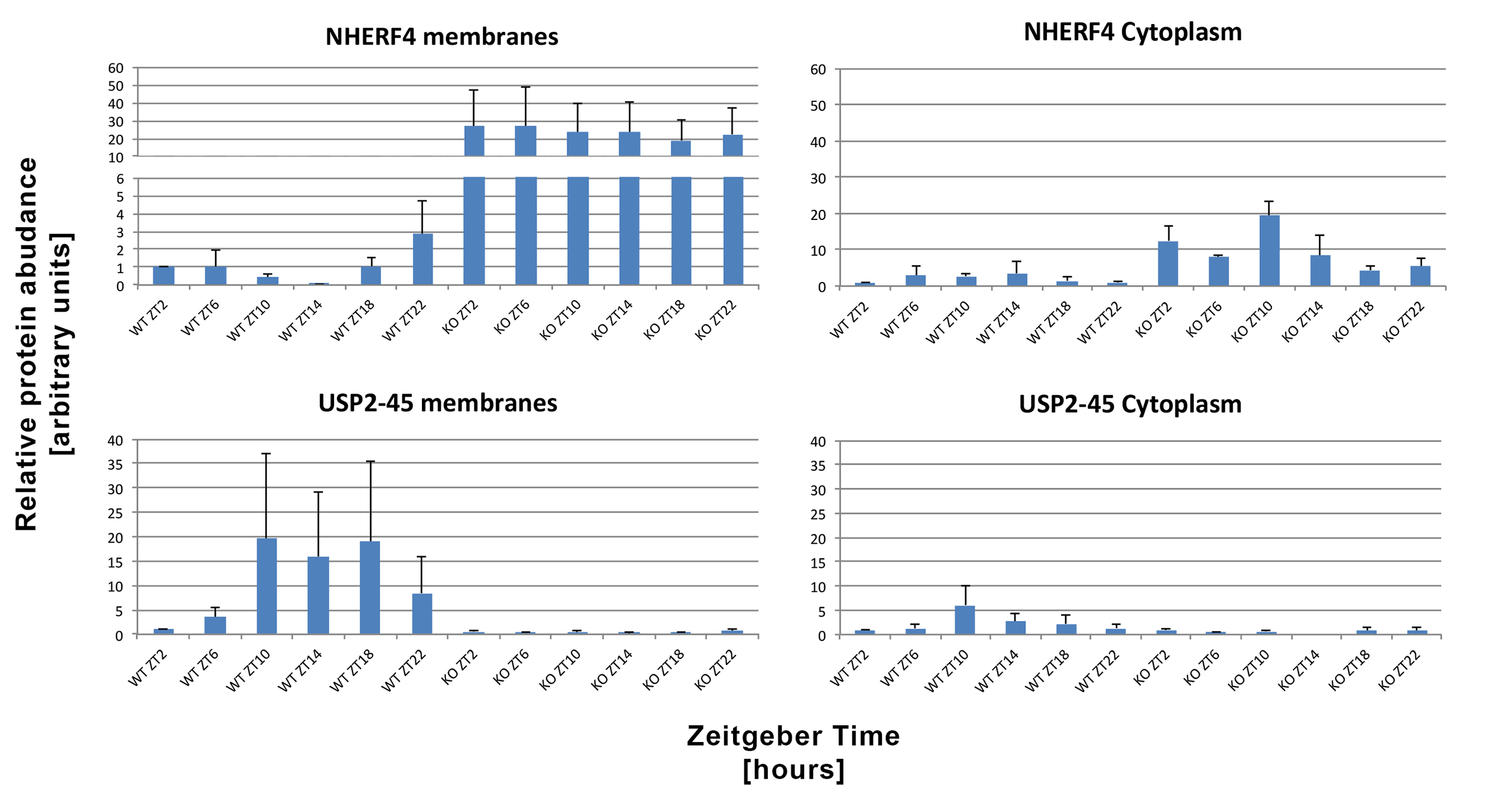

Supplement: S6 Fig — Western-blotting data shown on Fig 5 were quantified by densitometry. Data are presented as mean ± Standard Error of the Mean (SEM) of 2 independent experiments. (TIF) [file pone.0145155.s006.tif]

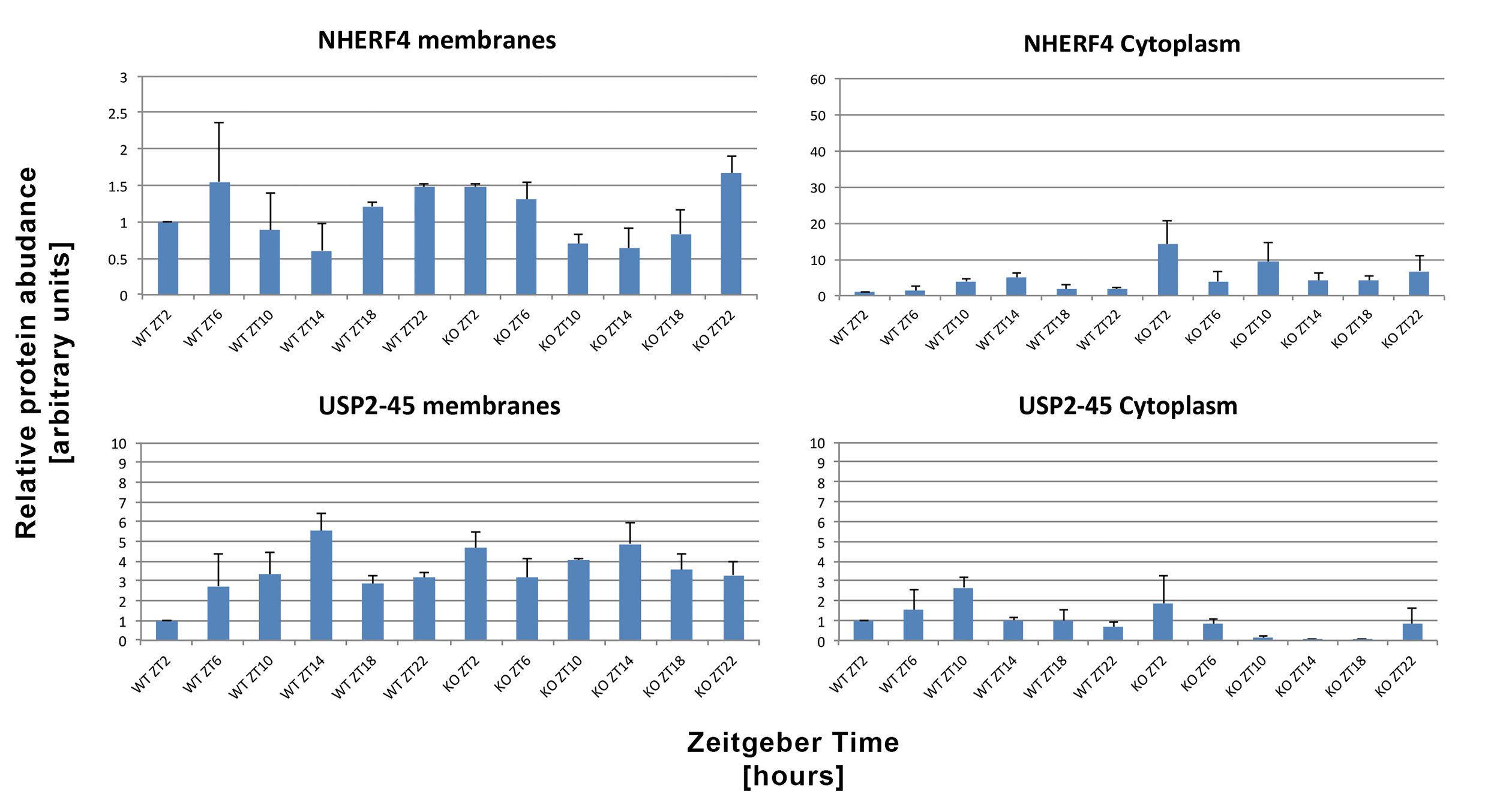

Supplement: S7 Fig — Western-blotting data shown in Fig 5 were quantified by densitometry. Data are presented as mean ± Standard Error of the Mean (SEM) of 2 independent experiments. (TIF) [file pone.0145155.s007.tif]
