# Supplementary material for: USP2-45 Is a Circadian Clock Output Effector Regulating Calcium Absorption at the Post-Translational Level
Source: PLoS One. 2016 Jan 12;11(1):e0145155. doi: 10.1371/journal.pone.0145155 (PMC4710524; doi:10.1371/journal.pone.0145155)
Supplement: S1 File — (PDF) [file pone.0145155.s008.pdf]

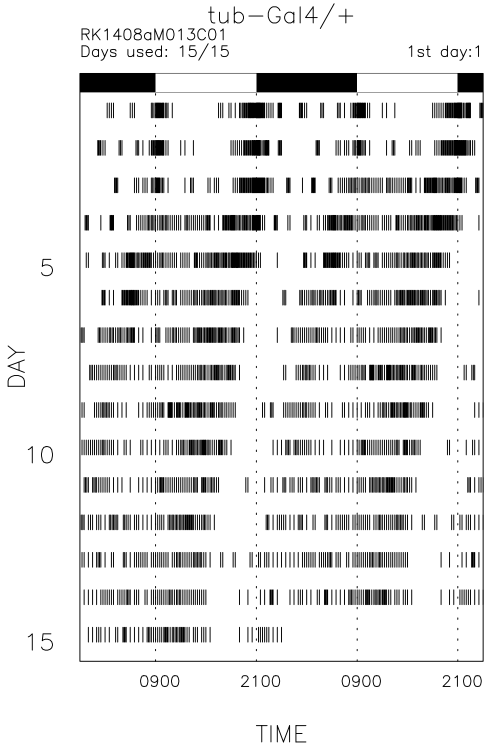

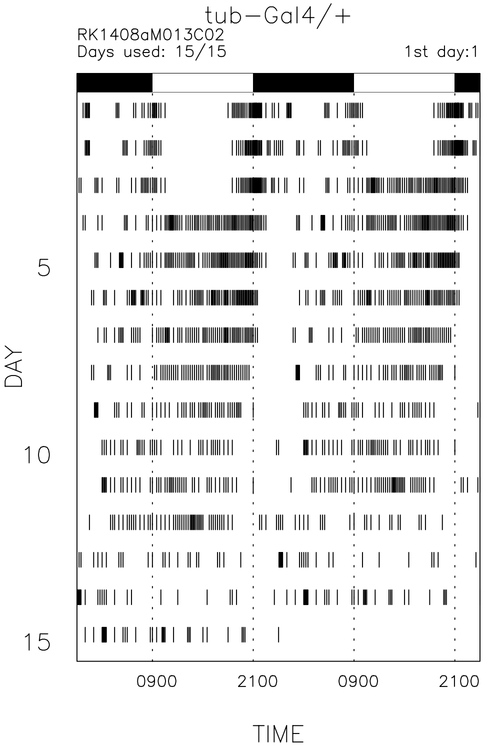

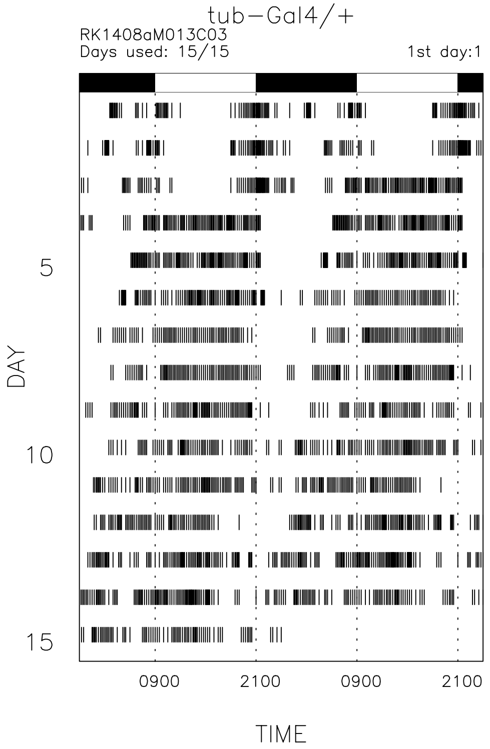

HashDensity: 12, Filter:Off, LightCycle: DD_2400, Light−On: 0900, Light−Off: 2100

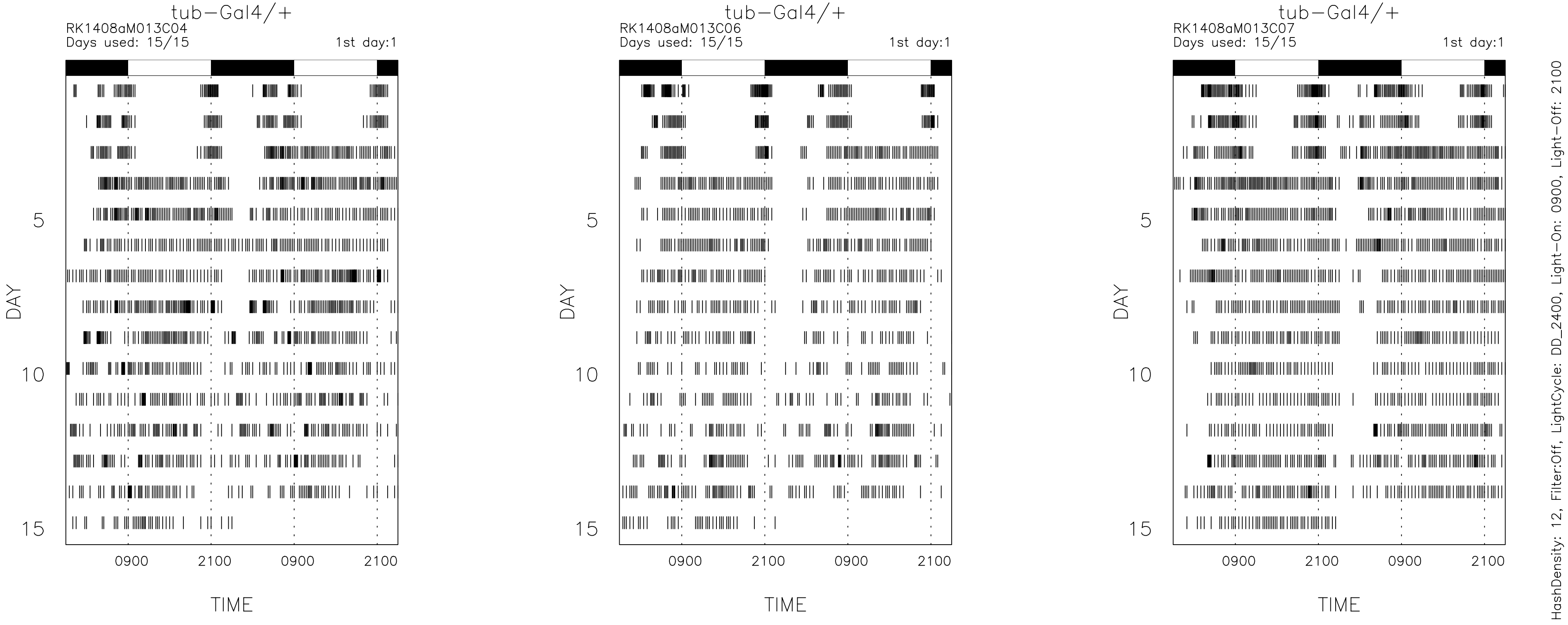

tub−Gal4/+
RK1408aM013C04
Days used: 15/15
1st day:1
DAY
5
10
15
0900
2100
0900
2100
TIME
tub−Gal4/+
RK1408aM013C06
Days used: 15/15
1st day:1
DAY
5
10
15
0900
2100
0900
2100
TIME
tub−Gal4/+
RK1408aM013C07
Days used: 15/15
1st day:1
DAY
5
10
15
0900
2100
0900
2100
TIME
HashDensity: 12, Filter:Off, LightCycle: DD_2400, Light−On: 0900, Light−Off: 2100

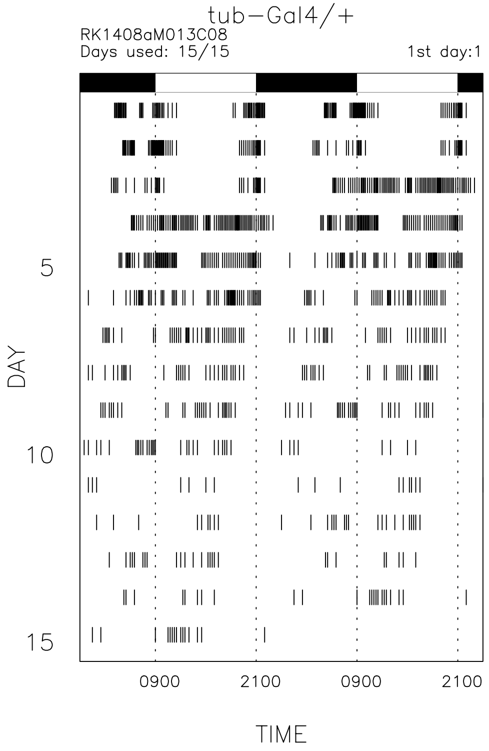

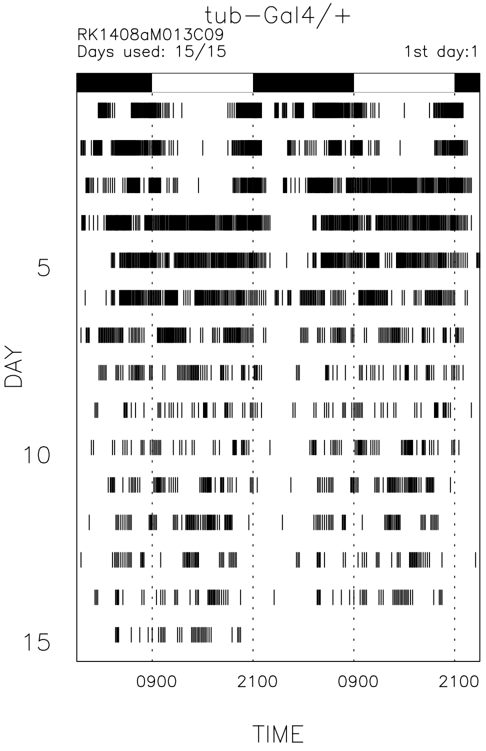

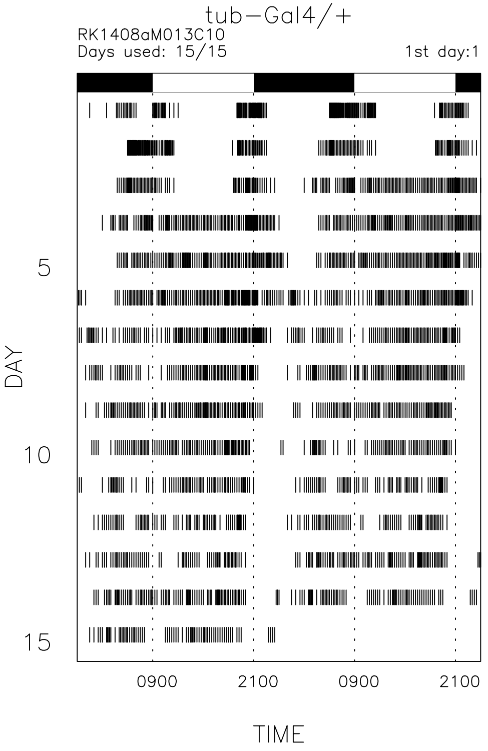

HashDensity: 12, Filter:Off, LightCycle: DD_2400, Light−On: 0900, Light−Off: 2100

tub−Gal4/+
RK1408aM013C11
Days used: 15/15 1st day:1

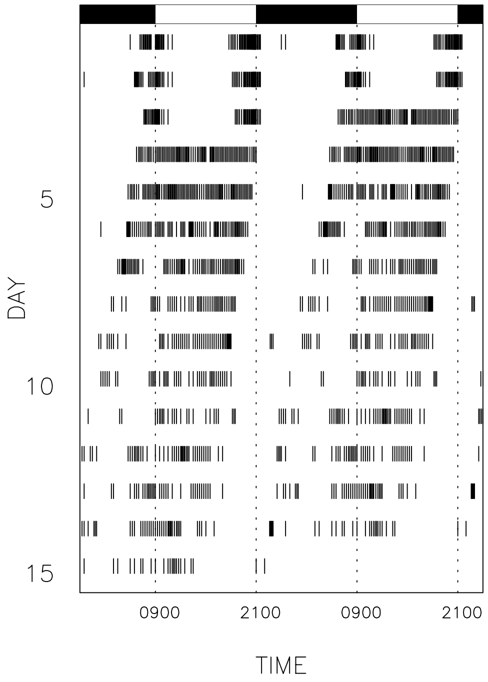

tub−Gal4/+
RK1408aM013C12
Days used: 15/15 1st day:1

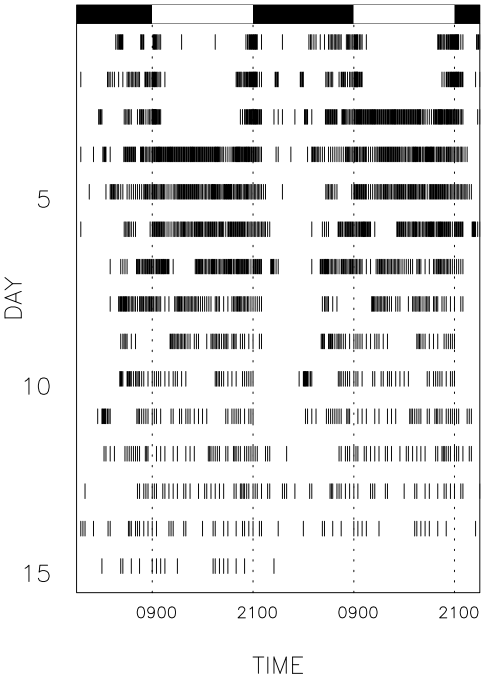

tub−Gal4/+
RK1408aM013C13
Days used: 15/15 1st day:1

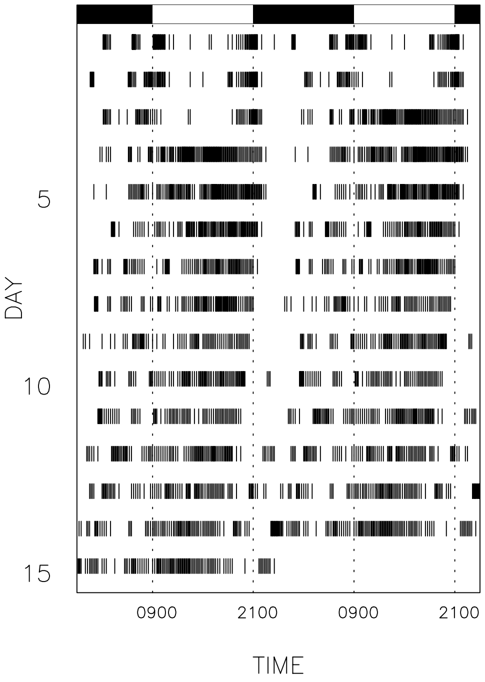

HashDensity: 12, Filter:Off, LightCycle: DD_2400, Light−On: 0900, Light−Off: 2100

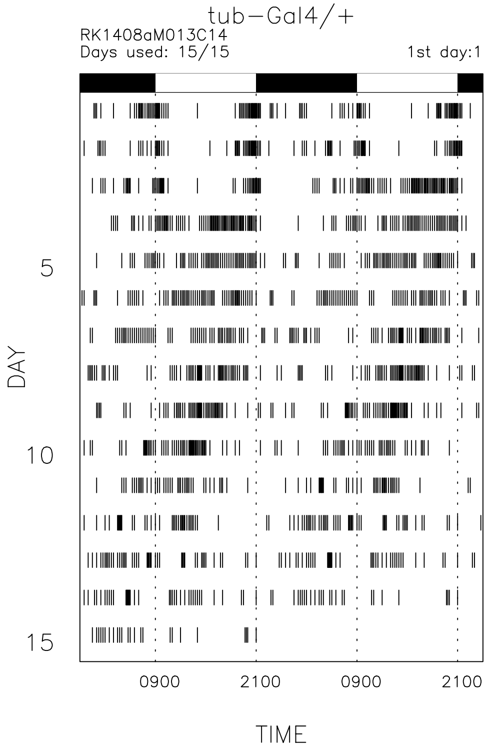

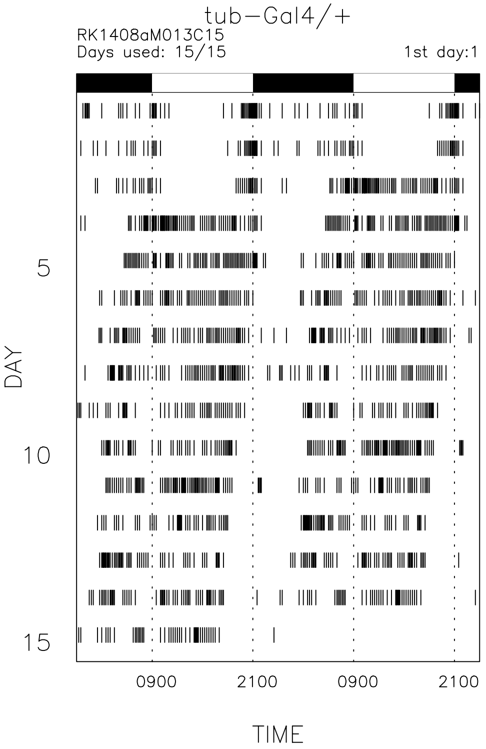

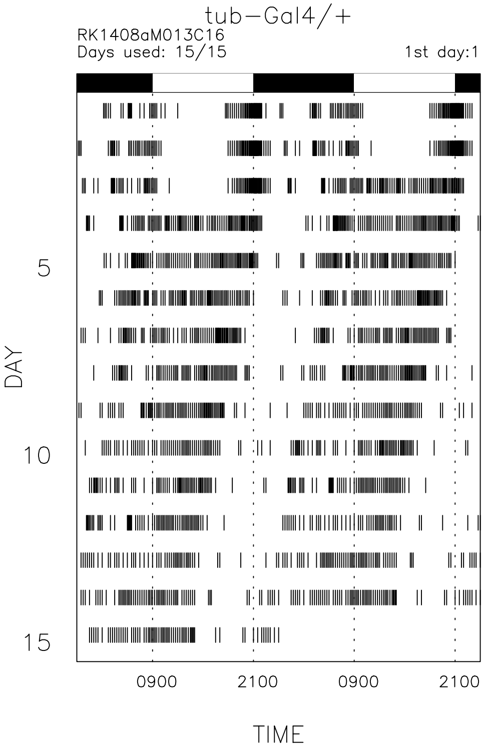

HashDensity: 12, Filter:Off, LightCycle: DD_2400, Light-On: 0900, Light-Off: 2100

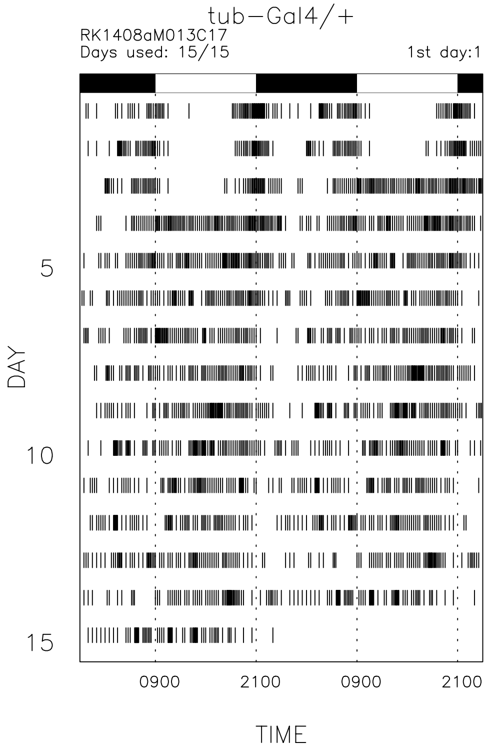

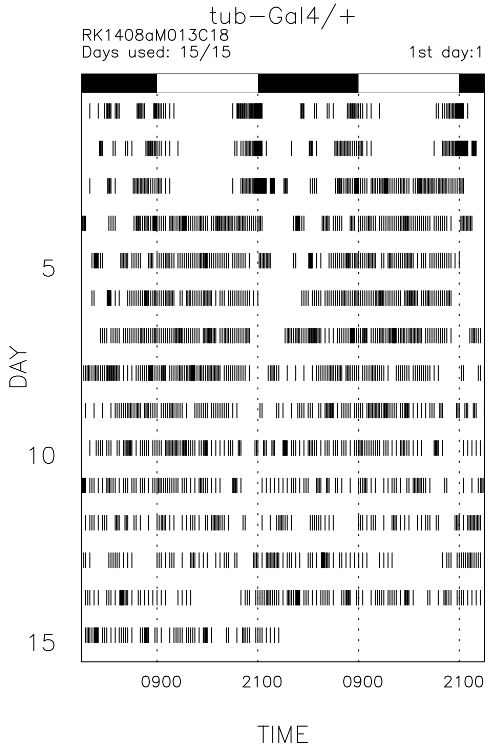

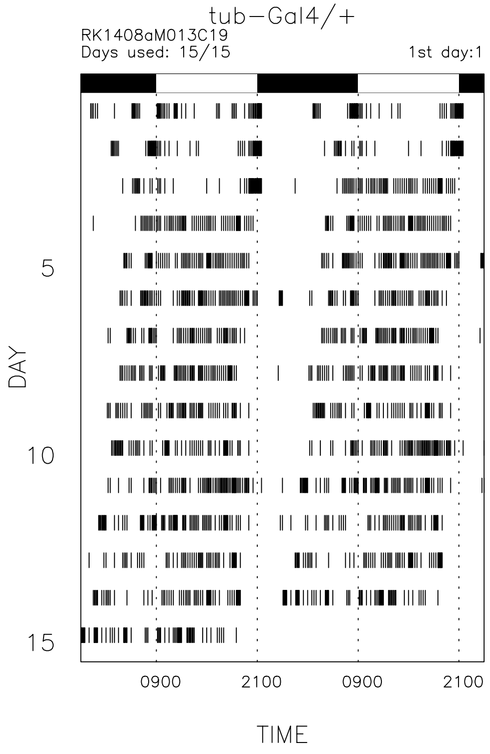

HashDensity: 12, Filter:Off, LightCycle: DD_2400, Light-On: 0900, Light-Off: 2100

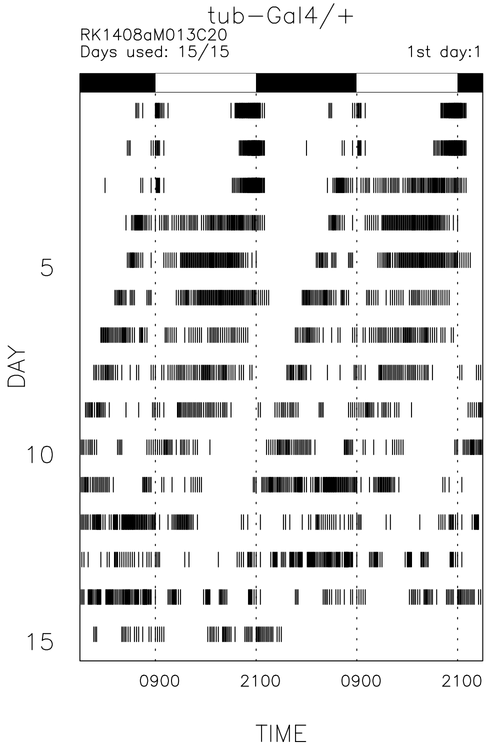

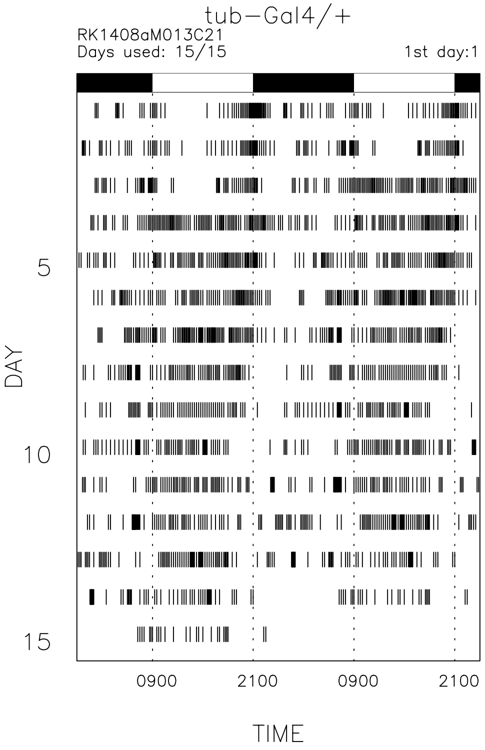

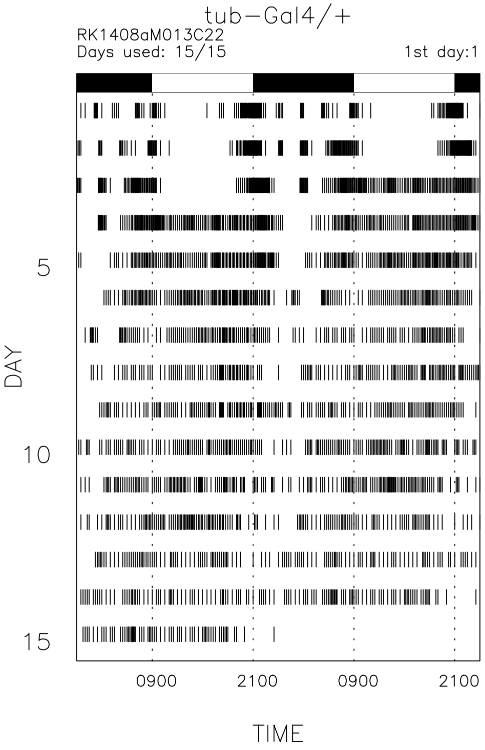

HashDensity: 12, Filter:Off, LightCycle: DD_2400, Light-On: 0900, Light-Off: 2100

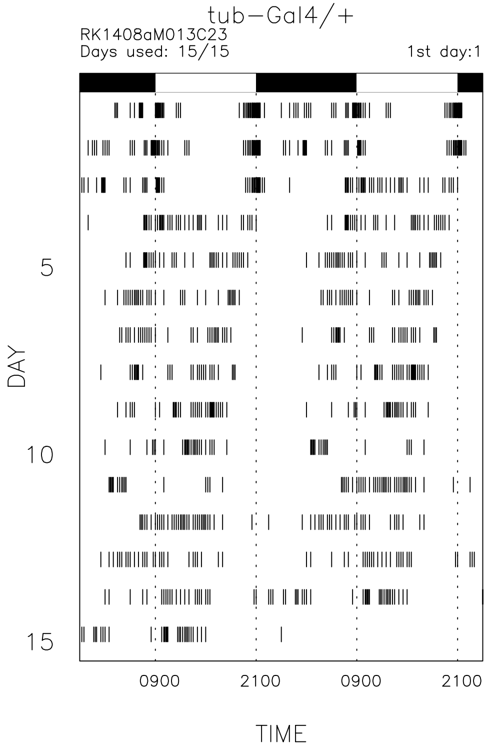

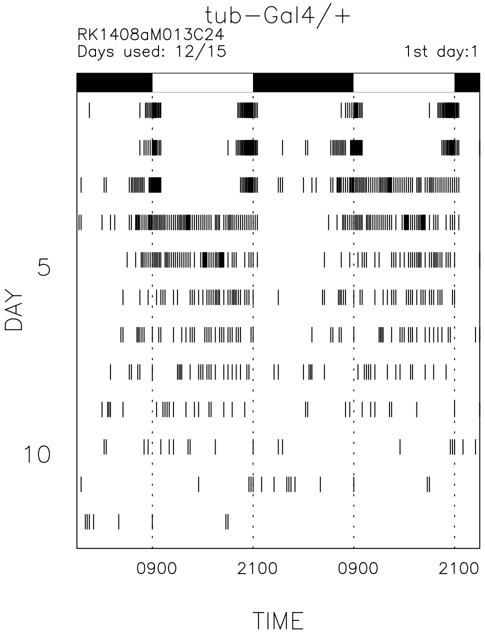

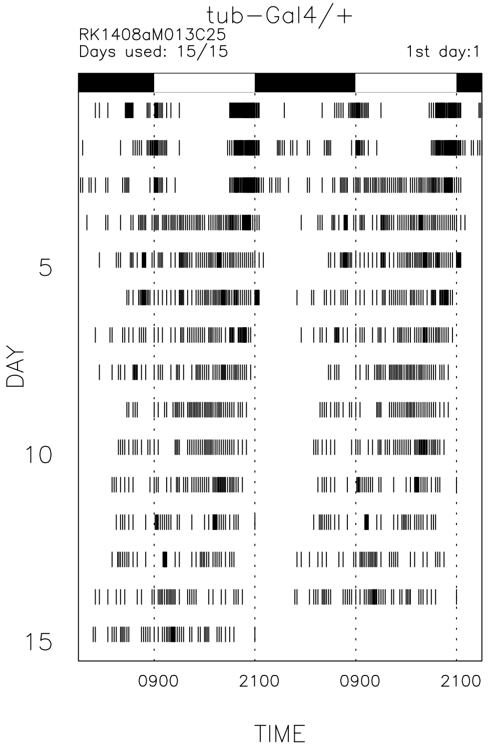

HashDensity: 12, Filter:Off, LightCycle: DD_2400, Light-On: 0900, Light-Off: 2100

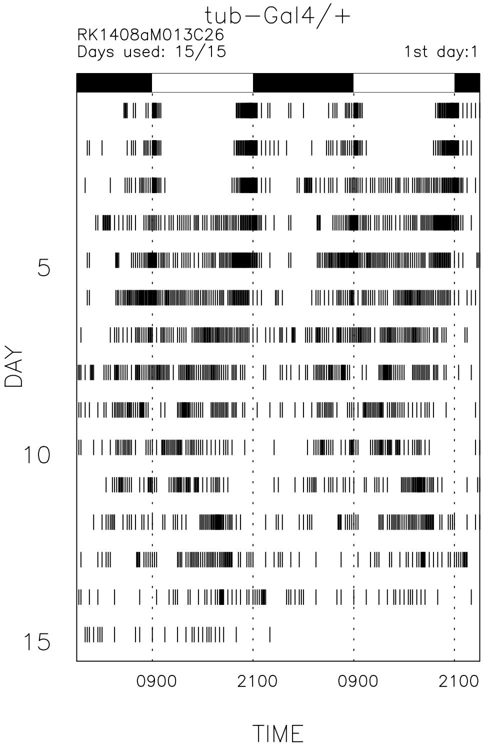

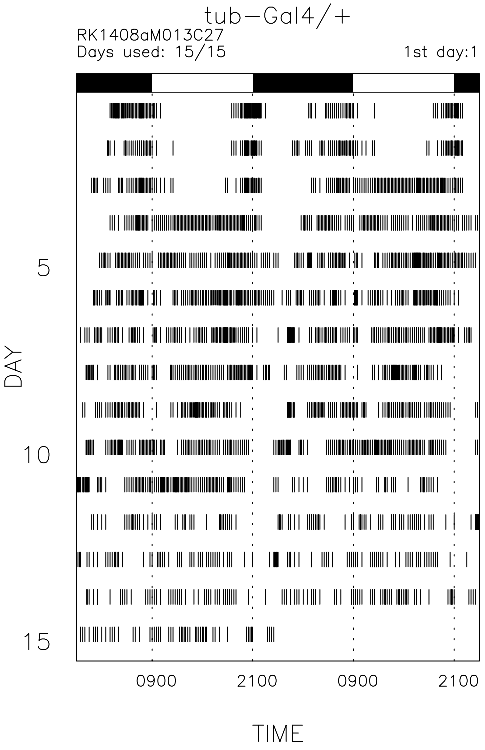

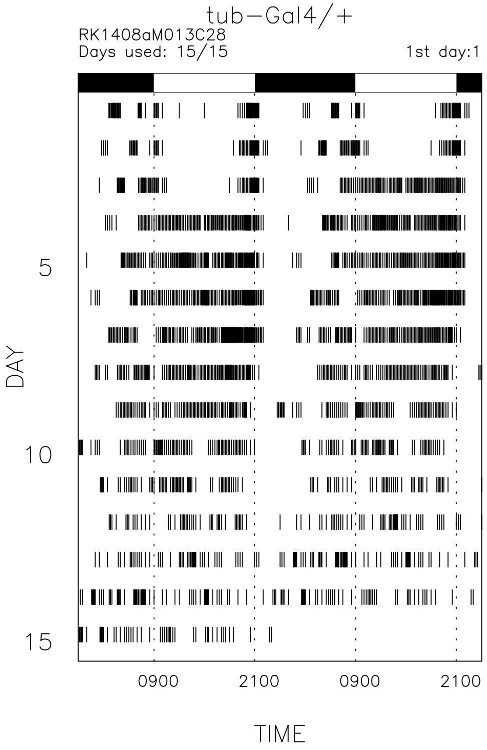

HashDensity: 12, Filter:Off, LightCycle: DD_2400, Light-On: 0900, Light-Off: 2100

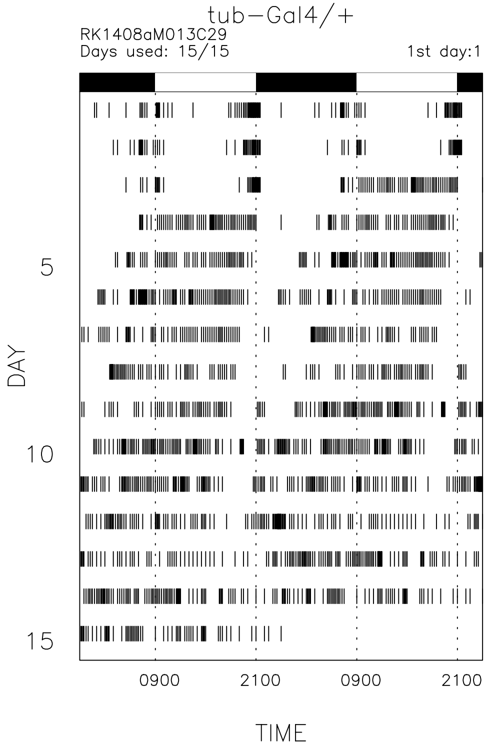

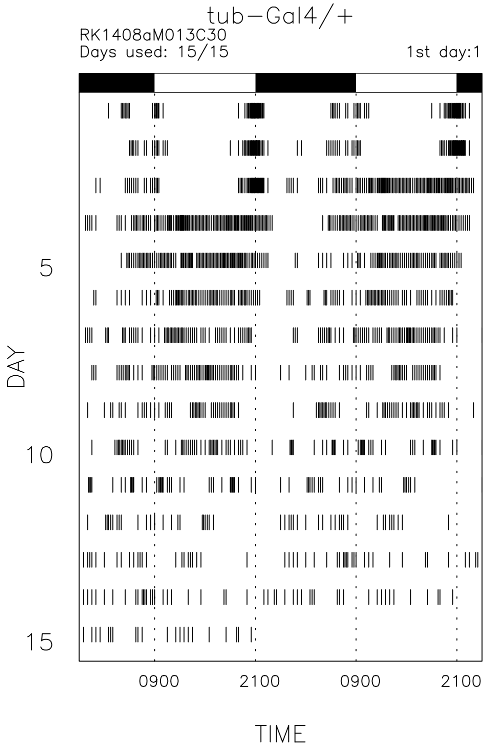

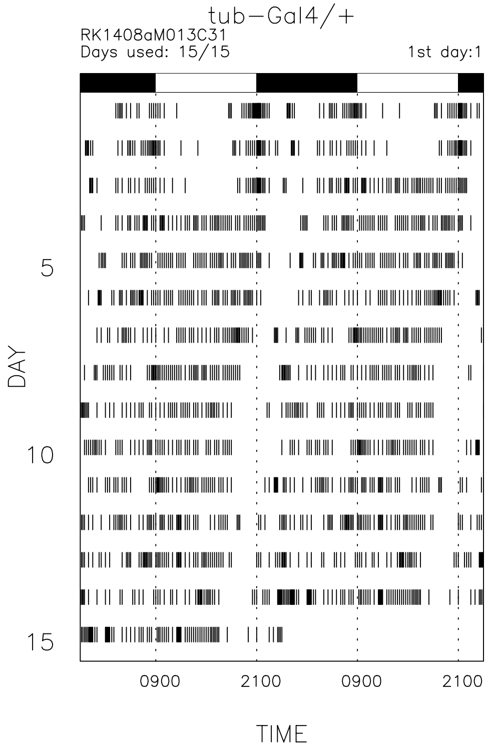

HashDensity: 12, Filter:Off, LightCycle: DD_2400, Light−On: 0900, Light−Off: 2100

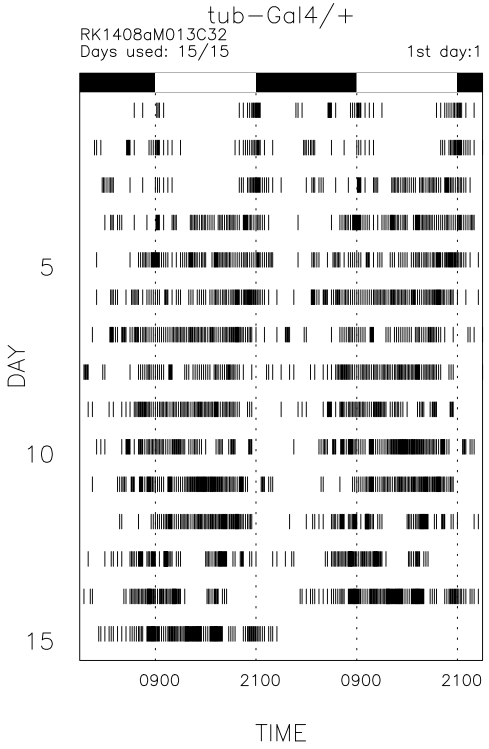

HashDensity: 12, Filter:Off, LightCycle: DD_2400, Light-On: 0900, Light-Off: 2100
