# Supplementary material for: USP2-45 Is a Circadian Clock Output Effector Regulating Calcium Absorption at the Post-Translational Level
Source: PLoS One. 2016 Jan 12;11(1):e0145155. doi: 10.1371/journal.pone.0145155 (PMC4710524; doi:10.1371/journal.pone.0145155)
Supplement: S2 File — (PDF) [file pone.0145155.s009.pdf]

tub>USP2 RNAi

RK1408aM048C01

Days used: 9/15 1st day:1

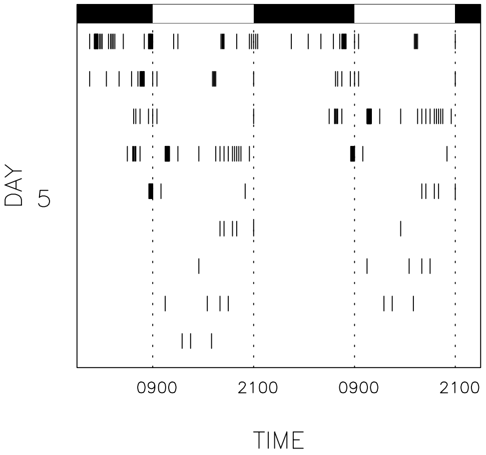

tub>USP2 RNAi

RK1408aM048C02

Days used: 15/15 1st day:1

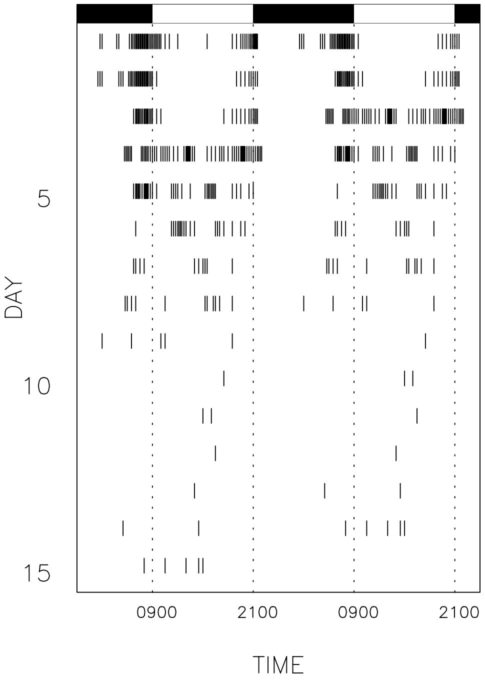

tub>USP2 RNAi

RK1408aM048C03

Days used: 15/15 1st day:1

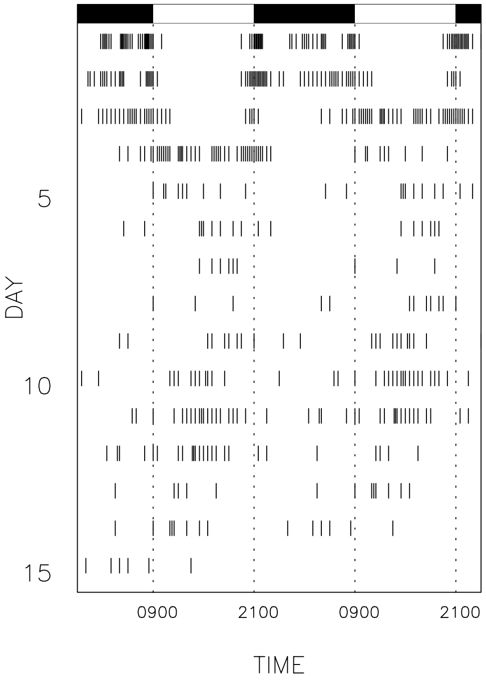

HashDensity: 12, Filter:Off, LightCycle: DD_2400, Light-On: 0900, Light-Off: 2100

tub>USP2 RNAi
RK1408aM048C04
Days used: 15/15 1st day:1

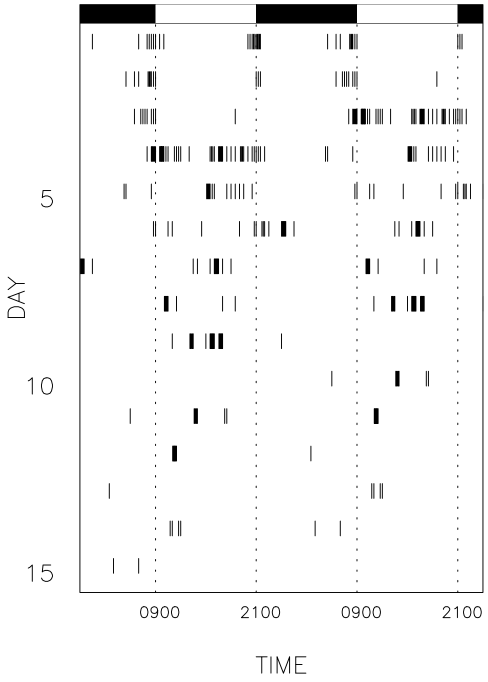

tub>USP2 RNAi
RK1408aM048C05
Days used: 10/15 1st day:1

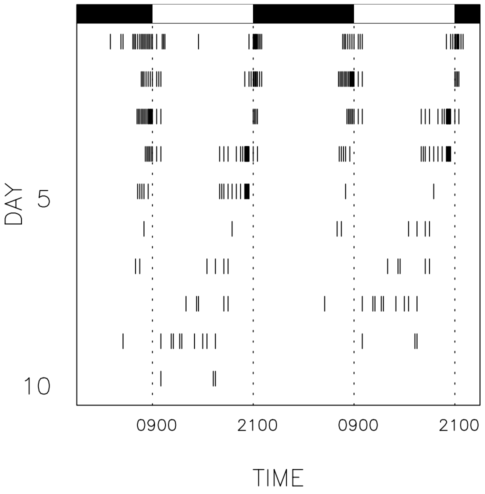

tub>USP2 RNAi
RK1408aM048C06
Days used: 13/15 1st day:1

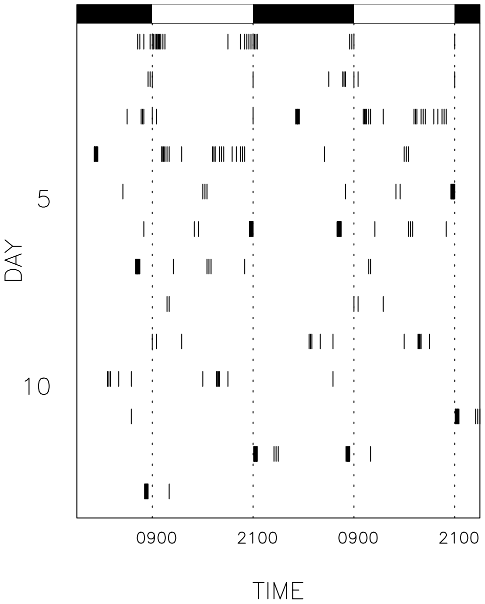

HashDensity: 12, Filter:Off, LightCycle: DD_2400, Light-On: 0900, Light-Off: 2100

tub>USP2 RNAi
RK1408aM048C07
Days used: 12/15 1st day:1

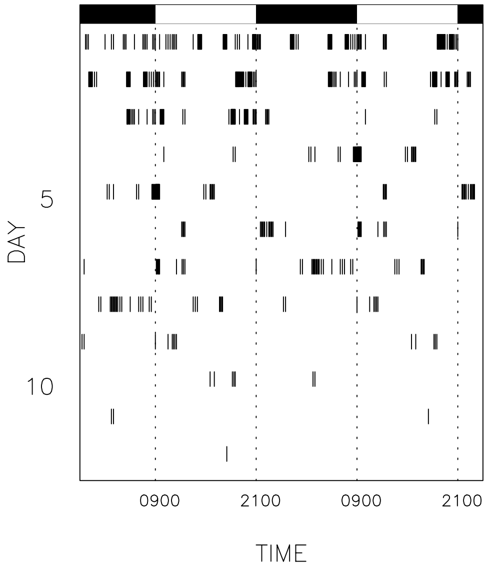

tub>USP2 RNAi
RK1408aM048C08
Days used: 9/15 1st day:1

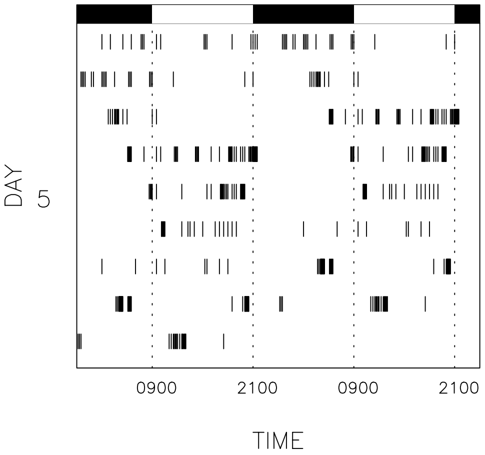

tub>USP2 RNAi
RK1408aM048C09
Days used: 3/15 1st day:1

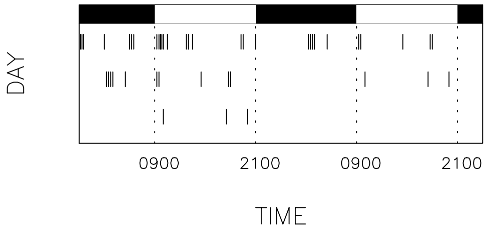

HashDensity: 12, Filter:Off, LightCycle: DD_2400, Light-On: 0900, Light-Off: 2100
