# Supplementary material for: USP2-45 Is a Circadian Clock Output Effector Regulating Calcium Absorption at the Post-Translational Level
Source: PLoS One. 2016 Jan 12;11(1):e0145155. doi: 10.1371/journal.pone.0145155 (PMC4710524; doi:10.1371/journal.pone.0145155)
Supplement: S1 Table — The femora of 9 Usp2-KO and 9 WT littermates were analysed by micro computed tomography (micro CT). Abbreviations: Full bone parameters (FULL): AVD: Apparent Volume Density; Cortical bone parameters (CORT): %BV: Cortical Bone Volume Density, Ct.Th: Cortical Thickness, J, Imax, Imin: Polar Moments of Inertia; Trabecular bone parameters (TRAB): BV/TV: Trabecular Bone Volume Density, BS/TV: Trabecular Bone Surface Density, BS/BV: Specific Bone Surface, Tb.Th: Trabecular Thickness. Tb.Sp: Trabecular Separation, Tb.N: Trabecular Number, Conn.D: Trabecular Connectivity Density. *: p<0.01 (PDF) [file pone.0145155.s010.pdf]

		FULL		CORT				
		**AVD**		**%BV**	**Ct.Th**	**J**	**Imax**	**Imin**
		**[%]**		**[%]**	**[µm]**	**[mm^4]**	**[mm^4]**	**[mm^4]**
WT	mean	**46.94**		**44.40**	**181.12**	0.40	0.27	0.13
	sd	**1.10**		**1.13**	**6.42**	0.03	0.02	0.01
Usp2-KO	mean	**44.04***		**42.22**	**174.29**	0.39	0.27	0.12
	sd	**1.30**		**2.32**	**7.79**	0.07	0.05	0.02

		TRAB						
		**BV/TV**	**BS/TV**	**BS/BV**	**Tb.Th**	**Tb.Sp**	**Tb.N**	**Conn.D**
		**[%]**	**[1/mm]**	**[1/mm]**	**[µm]**	**[µm]**	**[1/mm]**	**[1/mm^3]**
WT	mean	3.84	2.41	65.53	51.22	392.56	2.38	11.73
	sd	0.89	0.43	3.57	4.63	60.33	0.32	5.27
Usp2-KO	mean	3.77	2.41	64.44	50.88	368.04	2.42	14.76
	sd	1.16	0.68	4.79	1.21	51.33	0.43	9.84

Table S1

Pouly et al.
