# Supplementary material for: USP2-45 Is a Circadian Clock Output Effector Regulating Calcium Absorption at the Post-Translational Level
Source: PLoS One. 2016 Jan 12;11(1):e0145155. doi: 10.1371/journal.pone.0145155 (PMC4710524; doi:10.1371/journal.pone.0145155)
Supplement: S2 Table — The femora of 4 Usp2-KO and 6 WT littermates were analysed by micro computed tomography (micro CT). Abbreviations: Full bone parameters (FULL): AVD: Apparent Volume Density; Cortical bone parameters (CORT): %BV: Cortical Bone Volume Density, Ct.Th: Cortical Thickness, J, Imax, Imin: Polar Moments of Inertia; Trabecular bone parameters (TRAB): BV/TV: Trabecular Bone Volume Density, BS/TV: Trabecular Bone Surface Density, BS/BV: Specific Bone Surface, Tb.Th: Trabecular Thickness. Tb.Sp: Trabecular Separation, Tb.N: Trabecular Number, Conn.D: Trabecular Connectivity Density. (PDF) [file pone.0145155.s011.pdf]

		FULL		CORT				
		AVD [%]		%BV [%]	Ct.Th [µm]	J [mm^4]	Imax [mm^4]	Imin [mm^4]
WT	mean	41.66		1.33	63.61	49.13	547.05	1.73
	sd	1.55		0.22	3.56	2.52	61.89	0.18
Usp2-KO	mean	40.31		1.09	66.03	49.50	579.94	1.62
	sd	1.17		0.30	4.42	2.13	48.44	0.15

		TRAB						
		BV/TV [%]	BS/TV [1/mm]	BS/BV [1/mm]	Tb.Th [µm]	Tb.Sp [µm]	Tb.N [1/mm]	Conn.D [1/mm^3]
WT	mean	2.09	1.33	63.61	49.13	547.05	1.73	2.65
	sd	0.30	0.22	3.56	2.52	61.89	0.18	2.03
Usp2-KO	mean	1.65	1.09	66.03	49.50	579.94	1.62	2.79
	sd	0.43	0.30	4.42	2.13	48.44	0.15	2.34

Table S2

Pouly et al.
