# Supplementary material for: USP2-45 Is a Circadian Clock Output Effector Regulating Calcium Absorption at the Post-Translational Level
Source: PLoS One. 2016 Jan 12;11(1):e0145155. doi: 10.1371/journal.pone.0145155 (PMC4710524; doi:10.1371/journal.pone.0145155)
Supplement: S1 Text — (DOCX) [file pone.0145155.s012.docx]

**SUPPORTING INFORMATION**

*Protein sequences analysis.* Protein sequences of CG14619-PB (NP_728456.1), mUSP2-45 (NP_932759.1), mUSP21 (NP_038947.2), mUSP8 (NP_001239509.1) ad mUSP50 (AAH61020.1) were aligned with Clustal Omega default settings. Conserved Cys, QDE and His boxes of mUSP2, mUSP21, mUSP8 and mUSP50 [[1](#_ENREF_1)] were compared to homologous regions in CG14619-PB. Sequences identities were locally calculated in these three domains.

*Metabolic cages.* The animals were studied in metabolic cages similarly as described in the main material and methods section using control (0.61% Ca^2+^, Test Diet #5755, Richmond, USA) and low (0.02% Ca^2+^, Test Diet #5855, Richmond, USA) Ca^2+^ diets. Mice were maintained in LD and fed the 0.02% Ca^2+^ diet before being studied again for days 45 to 50. Plasma was collected at day 50 by tail vein incision for Ca^2+^ concentration measure. The animals were then fed the 0.02% Ca^2+^ diet for 4 more months and subsequently studied for bone structure following the micro-computed tomography protocol described hereafter.

## Urine and plasma analyses. Urine Mg^2+^, PO_4_^3-^ and creatinine were measured by the clinical chemistry lab of the Lausanne University Hospital (CHUV-LCC) using the Xylidyl Blue, Phosphomolybdate and Jaffe reaction methods, respectively.

*Plasma 25-(OH)-, 1,25-(OH)_2_-Vitamin D and PTH measurements*. Blood was collected in conscious mice by tail vein incision in Li^+^-Heparin coated tubes (Sarstedt, Nümbrecht, Germany). 25-(OH)- and 1,25-(OH)_2_-Vitamin-D were measured using a RIA (BL-29-CT and BL-47-CT respectively, BioLine, Brussels, Belgium) according to the manufacturer’s instructions. Intact (1-84) PTH was measured in plasma samples collected in K^+^-EDTA coated tubes (Sarstedt, Nümbrecht, Germany) by using an ELISA assay according to the manufacturer’s instructions (Alpco diagnostics, Salem, USA).

*Total protein extraction*. A segment of 3 cm downstream the stomach was isolated as the duodenum and washed with ice-cold PBS containing protease inhibitors cocktail (1 tablet/20ml; Roche) and 1mM PMSF. The mucosa was scraped-off from with a scalpel blade and snap frozen in liquid nitrogen. Total proteins were extracted as described for total liver extracts by [[2](#_ENREF_2)].

*Proteomic analyses.* A colony of 12 male *Usp2*-KO and WT littermates were entrained to LD and sacrificed by decapitation at ZT13 under red dim light. Duodenal mucosa was isolated as described above and quickly snap frozen in liquid nitrogen. Protein were extracted in a lysis buffer containing 8M Urea, 14 µg/ml Aprotinin, 0.7 µg /ml PepstatinA 0.7 µg /ml Leupeptin, 1 mM NaVO_4_, 10 mM Na-Pyrophosphate, 50 µM MG132 and 1mM PMSF and subsequently analyzed by the Protein Analysis Platform (PAF) of the University of Lausanne. Relative quantitation of proteins in 4 pools of 3 KO and 4 pools of 3 WT tissue samples was carried out by iTRAQ (Isobaric Tags for Relative and Absolute Quantitation) [[3](#_ENREF_3)] by the Protein Analysis Platform (PAF) of the University of Lausanne. After reduction and alkylation of cysteines, proteins were precipitated, redissolved in 8M Urea buffer and digested with trypsin (1:50 w:w) overnight. 80μg of digested material for each sample was labeled by reaction with one vial of iTRAQ 8-plex reagent (ABSciex) according to protocols supplied by the manufacturer. Samples were pooled, desalted and fractionated by off-gel electrofocusing as described [[4](#_ENREF_4)]. The 24 fractions obtained were analysed by nanoflow liquid chromatography-tandem mass spectrometry (nanoLC-MS/MS) on a hybrid linear trap LTQ-Orbitrap Velos mass spectrometer (Thermo Fisher Scientific, Bremen, Germany) on a two-hour gradient. Full MS survey scans were performed at 60’000 resolution and the ten most intense multiply charged precursor ions detected in the full MS survey scan were selected for HCD (Higher energy Collision Dissociation) fragmentation and Orbitrap analysis (7500 resolution). Each spectrum was acquired at two relative collision energies (35% and 45%) and the spectra were summed. Data files were analysed with MaxQuant 1.3.0.5 [[5](#_ENREF_5)] incorporating the Andromeda search engine [[5](#_ENREF_5)]. Further data analysis was performed using the R statistical programming language version 2.15.2 (R core team 2012). Intensities for the reporter ions 113 to 121 were normalized using the Variance Stabilizing method [[6](#_ENREF_6)] (R package version 3.26.0). Proteins with non-zero intensities in all channels were evaluated for differential expression using the Local-Pooled-Error method (R package version 1.32.0) [[7](#_ENREF_7)] followed by correction for multiple testing according to Benjamini and Hochberg [[8](#_ENREF_8)].

*Micro-computed tomography.* A colony of male *Usp2*-KO and WT littermates were maintained under standard housing chow or 0.02% Ca^2+^ diet in LD until the age of 12 months. The animals were sacrificed by cervical dislocation after irreversible anaesthesia (0.8 mg Xylazine and 1 mg Ketamine per kg of body weight in 0.9% NaCl injected intraperitoneally). Femora were dissected immediately stored at 4°C in 70% Ethanol. Micro-computed tomography was performed by B-cube AG (Brüttisellen, Switzerland) as described in [[9](#_ENREF_9)].

**SUPPLEMENTARY REFERENCES**

1. Quesada V, Diaz-Perales A, Gutierrez-Fernandez A, Garabaya C, Cal S, et al. (2004) Cloning and enzymatic analysis of 22 novel human ubiquitin-specific proteases. Biochem Biophys Res Commun 314: 54-62.

2. Cretenet G, Le Clech M, Gachon F (2010) Circadian clock-coordinated 12 Hr period rhythmic activation of the IRE1alpha pathway controls lipid metabolism in mouse liver. Cell Metab 11: 47-57.

3. Ross PL, Huang YN, Marchese JN, Williamson B, Parker K, et al. (2004) Multiplexed protein quantitation in Saccharomyces cerevisiae using amine-reactive isobaric tagging reagents. Mol Cell Proteomics 3: 1154-1169.

4. Geiser L, Dayon L, Vaezzadeh AR, Hochstrasser DF (2011) Shotgun proteomics: a relative quantitative approach using Off-Gel electrophoresis and LC-MS/MS. Methods Mol Biol 681: 459-472.

5. Cox J, Mann M (2008) MaxQuant enables high peptide identification rates, individualized p.p.b.-range mass accuracies and proteome-wide protein quantification. Nat Biotechnol 26: 1367-1372.

6. Huber W, von Heydebreck A, Sultmann H, Poustka A, Vingron M (2002) Variance stabilization applied to microarray data calibration and to the quantification of differential expression. Bioinformatics 18 Suppl 1: S96-104.

7. Jain N, Thatte J, Braciale T, Ley K, O'Connell M, et al. (2003) Local-pooled-error test for identifying differentially expressed genes with a small number of replicated microarrays. Bioinformatics 19: 1945-1951.

8. Benjamini Y, Hochberg Y (1995) Controlling the false discovery rate: a practical and powerful approach to multiple testing. J R Stat Soc Ser B 57: 289-300.

9. Kohler T, Stauber M, Donahue LR, Muller R (2007) Automated compartmental analysis for high-throughput skeletal phenotyping in femora of genetic mouse models. Bone 41: 659-667.
